# Alternatives to animal-derived extracellular matrix hydrogels? An explorative study with HepaRG cells in animal-free hydrogels under static and dynamic culture conditions

**DOI:** 10.3389/ftox.2025.1649393

**Published:** 2025-10-30

**Authors:** Katharina S. Nitsche, Paul L. Carmichael, Sophie Malcomber, Iris Müller, Hans Bouwmeester

**Affiliations:** ^1^ Division of Toxicology, Wageningen University, Wageningen, Netherlands; ^2^ Unilever Safety, Environmental and Regulatory Science (SERS), Colworth Science Park, Sharnbrook, United Kingdom

**Keywords:** new approach methodologies, microphysiological systems, organ-on-chip, 3R principle, next-generation risk assessment

## Abstract

New approach methodologies (NAMs) aim for animal-free chemical risk assessments. However, many *in vitro* NAM models still depend heavily on Matrigel and collagen despite the ethical, reproducibility, and biomedical concerns regarding the use of animal-derived materials. As awareness regarding this problem grows, several animal-free extracellular matrix hydrogel alternatives have emerged in the market. However, NAM studies with alternative hydrogels are rather scarce. The present study provides a concise review of commercially available animal-free hydrogels as well as an experimental screening approach to identify biocompatible candidates for HepaRG cell culturing under static and dynamic conditions in a 96-well plate and an OrganoPlate 3-lane device (Mimetas B.V.), respectively. The hydrogels evaluated herein include PeptiMatrix Core and PuraMatrix as synthetic peptides, VitroGel Organoid-3 as a synthetic polysaccharide, GrowDex as a wood-derived polysaccharide, and a Matrigel-collagen mix as the animal-derived reference. The health and functionality of the HepaRG cells were assessed via viability, lactate dehydrogenase leakage, albumin and bile acid secretion, CYP3A4 enzyme activity, and gene expression analyses. All animal-free hydrogels tested herein supported HepaRG cell proliferation in both culture conditions, although the cells had inadequate structure support and exhibited lower hepatic synthetic capacity in the OrganoPlate microphysiological system device. Notably, the cells grown in PeptiMatrix 7.5 showed promising metabolic competence under perfusion, making it a potential candidate for xenobiotic metabolism studies after further optimisation. These findings serve as a starting point to encourage scientists to take steps towards more animal-free cell culturing.

## 1 Introduction

New approach methodologies (NAMs) such as 3D cell culturing and microphysiological systems (MPSs) strive to comply with the 3R principle of replacement, reduction, and refinement of animals in scientific procedures. However, many *in vitro* NAMs still rely heavily on animal-derived materials for cell culturing, including foetal bovine serum (FBS), bovine serum albumin (BSA), trypsin, and purified extracellular matrix (ECM) hydrogels for coating and scaffolding (van der Valk, 2018; Oredsson et al., 2019). The most commonly used ECMs are collagen (type Ⅰ) and Matrigel that are derived from animal materials, making them complex and poorly defined basement membrane extracts with high lot-to-lot variability. Because these materials are often sourced from multiple animals, the composition involving protein content, growth factors, and other bioactive molecules can vary dramatically between batches. This variability in composition can alter cell growth, differentiation, and treatment responses, ultimately reducing the experimental reproducibility and limiting the suitability of the created test models. Despite the limitations regarding the ethics of use and reproducibility, Matrigel and type I collagen remain the most selected options for cell culture applications (Kleinman and Martin, 2005; Aisenbrey and Murphy, 2020). However, there is a growing emergence of manufacturers offering animal-free ECM alternatives with the promise of functional cell support, reproducibility, reliability, robustness, and human relevance, similar to the promise of MPS devices.

MPS models entail small scale devices offering cells a microenvironment of a specific tissue or organ to mimic the physiological aspects important for their functioning or pathophysiological condition (Humayun et al., 2021). These systems were developed to improve upon traditional 2D cell systems. Studies performed under dynamic culture conditions using MPS technologies emphasise that the interplay between flow and 3D architecture can critically influence cell mechanobiology, impacting not only the cytoskeleton but also the cellular signalling pathways driving proliferation, maturation, and long-term cell functioning (Ohashi et al., 2017). However, this physiologically relevant shift in cell culturing from static to dynamic and from 2D to 3D relies on the support of ECM hydrogels, which are necessary for coating and scaffolding. In MPS devices, the ECM hydrogels enable cells to properly attach, grow, and differentiate as well as provide a physical barrier between cell types and/or between culture compartments in some cases (Jensen and Teng, 2020; Nitsche et al., 2022). The need for a physical barrier is especially applicable to closed MPS devices like the OrganoPlate that was also used in the present study. This MPS device requires an ECM hydrogel for chip seeding to culture cells in suspension and/or against a gel (e.g., tubule) as well as create an environment that guides the gravity-driven flow of the medium (Nitsche et al., 2022).

*In vivo*, the ECM is essential for structurally and biologically supporting the cells in tissues. The ECM is composed of three main fibrous proteins, namely, collagen (types Ⅰ, ⅠⅠ, ⅠⅠⅠ, Ⅴ, and Ⅺ), non-collagens (e.g. fibronectin and entactin), and glycoproteins (e.g., laminin and perlecan), as well as proteins for integrins, growth factors, and enzymes (e.g., matrix metalloproteinases) (Kular et al., 2014). The ECM composition is not only tissue-specific but also constantly remodelled either enzymatically or non-enzymatically, with the molecular components being continuously subjected to post-translational modifications (Frantz et al., 2010; Nawaz et al., 2018). In contrast, *in vitro* cell culture ECMs like collagen types Ⅰ and ⅠⅤ are composed of one major component, whereas Matrigel contains approximately 60% laminin, 30% collagen, and 8% entactin along with traces of perlecan (Corning Inc, 2023). The animal-free ECM hydrogel alternatives have simpler compositions and are either derived naturally (e.g. fibrillar cellulose and agarose) (Hickey and Pelling, 2019) or synthetically (e.g., peptides and polysaccharides). Synthetic hydrogels are designed to mimic single or multiple ECM proteins through a peptide base with a natural protein structure to support functional cell and tissue interactions (Neves et al., 2020; Feodoroff et al., 2023). Unlike the popular options Matrigel and collagen, synthetic and natural hydrogels can often be tuned independently in terms of their mechanical and biological properties to fit the research context by the addition of (synthetic) growth factors, the adjustment of crosslinker concentrations, the adjustment of density for stiffness, or polymerisation (Caliari and Burdick, 2016; Li et al., 2018; Aisenbrey and Murphy, 2020; Charbonier et al., 2021). Additionally, synthetic and natural hydrogels have potentially lower nonspecific binding of (lipophilic) chemicals depending on their compositions (e.g., hydrophobicity of biomolecules), charge interactions with xenobiotics, roughness, or porosity to trap molecules. However, a broader adoption of animal-free ECM alternatives remains scarce (Aisenbrey and Murphy, 2020; Kozlowski et al., 2021; Duarte et al., 2023).

In this study, we evaluated commercially available animal-free hydrogels with the aim of identifying and comparing their abilities to maintain viable and functional HepaRG cultures in both static and dynamic environments. The study followed a three-tiered approach. First, we reviewed literature to identify hydrogel candidates that cover a broad spectrum of major components and physiochemical properties for our pre-screening. Next, we pre-screened hydrogels in 48-well plates for their biocompatibility to support viable and growing cultures, as well as their ability for uptake by the MPS device. Lastly, we differentiated HepaRG cells *in situ* to monitor the key functional characteristics and assess the relative gene expressions in 96-well plates and the OrganoPlate 3-lane MPS device. The hepatoma cell line HepaRG was used as reference cell line, given its unique differentiation ability into metabolically active liver cells, especially under dynamic culture (Ramaiahgari and Ferguson, 2019; Duivenvoorde et al., 2021; Waizenegger et al., 2021; Stanley and Wolf, 2022). We used several key markers to evaluate the cell health and synthetic capacity under both static and dynamic conditions, namely viability, lactate dehydrogenase (LDH) leakage, basal albumin secretion, primary (conjugated) bile acid production, and CYP3A4 enzyme activity. For comparative analyses with the reference Matrigel-collagen culture under both conditions, we included quantitative polymerase chain reaction (qPCR) for selected target genes, including albumin, CYP3A4, bile acid relevant CYP27A1 and CYP7B1, as well as cytoskeletal markers KRT18 (hepatocytes) and KRT19 (cholangiocyte-like cells). Finally, we evaluated the on-chip cell population and distribution through immunofluorescent staining.

## 2 Materials and methods

### 2.1 Literature review

This study includes a concise literature review summarising the commercially available hydrogels and their major components to aid selection of hydrogels for testing and to understand any differences in the observed results. The literature search was first conducted in PubMed and Scopus using general keywords like ‘animal-free cell culture’ and ‘animal-free hydrogels’. In addition, separate searches were conducted for each major hydrogel component by combining the terms ‘[major component] cell culture’ (e.g. synthetic peptide cell culture) to collect application-specific studies. To complement these searches, we screened manufacturer websites for product-specific protocols and application notes. The inclusion criteria were peer-reviewed studies and manufacturer-provided information published between 1980 and October 2024. The selected articles and manufacturer data were further screened for details regarding the physical characteristics, technical considerations (e.g. lab handling), and cell-culture applications.

### 2.2 MPS device and chemicals

**FIGURE 1:**
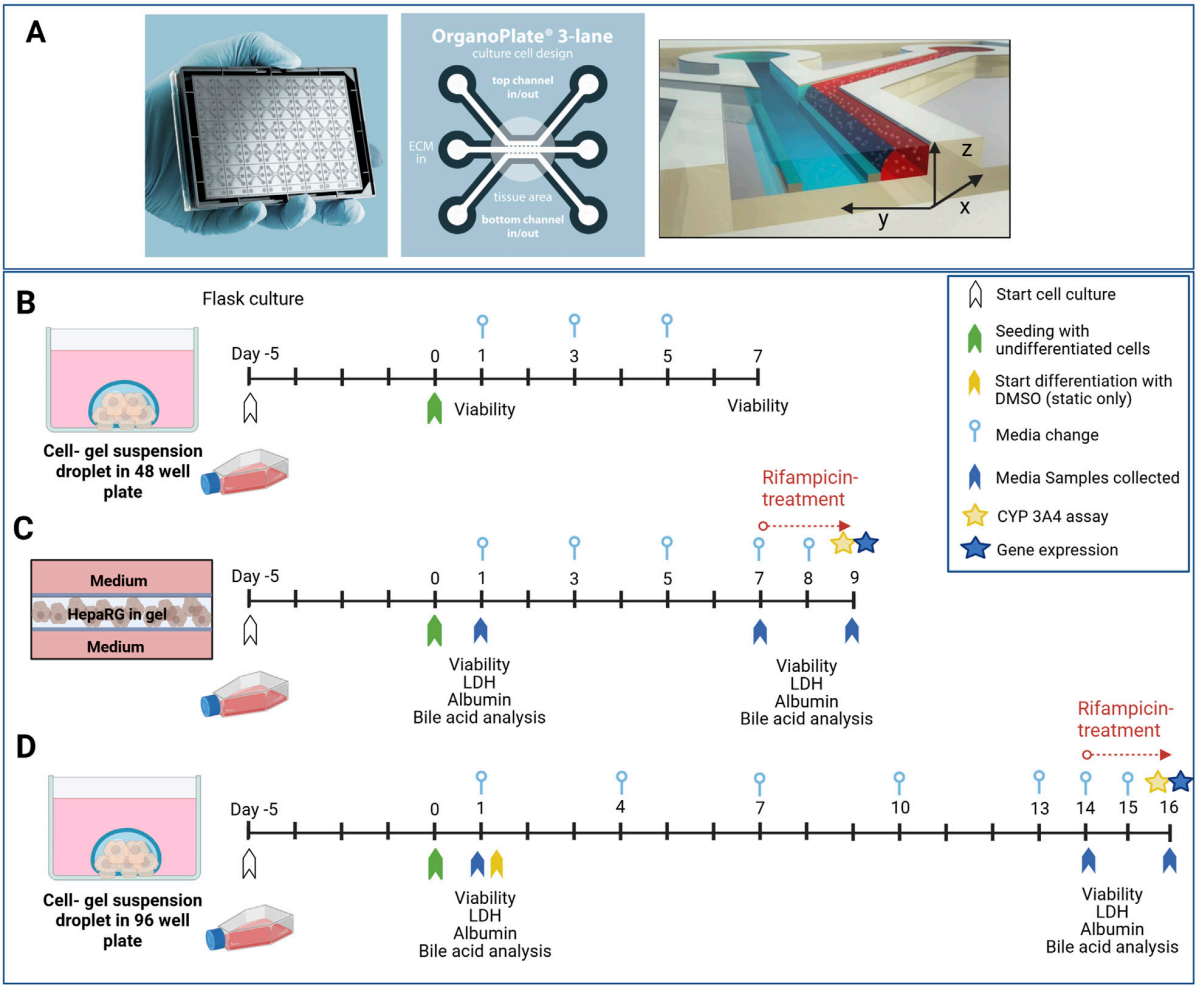
HepaRG culture setups under static and dynamic conditions. **(A)** The OrganoPlate 3-lane 40 device (Mimetas B.V., Leiden, Netherlands) consists of two perfusion channels (top and bottom) and a central ECM channel that are separated by PhaseGuides. **(B–D)** Experimental timelines are shown for pre-screening and in-depth evaluation of the different hydrogels under static and dynamic conditions. Day 0 marks the seeding of undifferentiated HepaRG cells in gel on 96-well plates and on the OrganoPlate. **(B)** Pre-screening for viability support on a 48-well plate over 7 days. **(C)** The OrganoPlate 3-lane study spanned 9 days, including 48 h of rifampicin treatment from day 7 (25 μM, 0.1% dimethylsulfoxide (DMSO)). The medium was changed on alternate days, and samples were collected for the secretome analysis on days 1, 7, and 9. **(D)** The static 96-well plate study design spanned 16 days, involving a gradual increase in DMSO for HepaRG differentiation and 48 h of rifampicin treatment (25 μM, 0.1% DMSO) from day 14. The medium was changed every third day to minimise gel disturbance, and samples were collected on days 1, 14, and 16.

The dynamic experiments were performed with the OrganoPlate^®^ 3-lane device (MIMETAS 4004B-400B, Leiden, Netherlands) that utilises a 384-well plate format embedding 40 culture chamber chips, two perfusion channels, and one ECM channel. The channels are separated by PhaseGuides™ (100 × 55 μm, width × height; Figure 1), which are microfabricated passive barriers acting as ‘liquid pinning’ structures that leverage surface tension and capillary forces to confine the ECM to defined regions (Mimetas, 2022). Perfusion is driven by gravity and induced using OrganoFlow^®^ S at the recommended 7° rocking angle and 8 min interval with intermittent shear stress forces ranging between 0 and 1.41 dyne/cm^2^ (Mimetas, 2022). Rifampicin powder (Vetranal^®^, Supelco, Sigma-Aldrich, Zwijndrecht, Netherlands) was dissolved in dimethylsulfoxide (DMSO; Sigma-Aldrich, CAS 67-68-5) for the CYP3A4 enzyme induction studies.

### 2.3 Cell culture

In this work, we prioritised isolation of the matrix variable under standard culture conditions (10% FBS) to limit further confounding and to attribute the effects to the ECM hydrogels. However, we recognise that the undefined and batch-variable composition of FBS can significantly influence the cell behaviour, phenotype, and assay outcomes and should therefore be replaced in future studies. The HepaRG cells (Biopredic International, Rennes, France) were cultured in a growth medium consisting of 500 mL of Williams E culture medium (Gibco, Thermo Fisher, Paisley, United Kingdom), 5 mL of penicillin–streptomycin solution (1% v/v, Thermo Fisher Scientific), 5 mL of L-glutamine (1% v/v, Thermo Fisher Scientific), 0.25 mL of human insulin (0.05% v/v, 5 µg/mL, Sigma-Aldrich), 50 mL of FBS (10% v/v, Sigma-Aldrich), and 5 mL of hydrocortisone-21-hemisuccinate (10 mg/mL, 1% v/v, Sigma-Aldrich). Before seeding in different matrices, the undifferentiated HepaRG cells were maintained in T75 tissue culture flasks (Greiner Bio-One B.V., Netherlands) according to the manufacturer’s instructions and subcultured at 85% confluency. Passages 17–21 were used for the static culture and chip seeding. The medium in the flasks was replaced 2–3 times a week after washing with phosphate-buffered saline (PBS; Gibco, Thermo Fisher) and maintained under humidified incubation (37 °C, 5% CO_2_). For subculturing and seeding, the cells were dissociated with trypsin-EDTA (trypsin 0.025%/EDTA 0.01%; Invitrogen, Thermo Fisher Scientific, Breda, Netherlands).

### 2.4 Cell culture for pre-screening of hydrogels

Four hydrogels were pre-screened under the static condition on 48-well plates (Greiner Bio-One B.V., Germany) to evaluate their biocompatibilities for viable cell cultures over 7 days (Figure 1B). To allow comparisons among the hydrogels, the seeding conditions were standardised. Briefly, the HepaRG cells cultured in flasks were dissociated, collected in medium, and counted using a Cellometer^®^ Auto T4 bright-field cell counter (Nexcelom Bioscience), before being pelleted (100×*g* for 5 min) and later encapsulated in gel. A total volume of 80 μL of the cell-gel suspension was seeded with 25,000 cells per well to enable the cells to self-organise in the matrix. Depending on the protocol (described below), 250 µL of the culture medium was added either immediately to promote gel stabilisation or after the gel had stabilised. PeptiMatrix Core (12.5 mg/mL; PeptiMatrix™, Nottingham, United Kingdom; hereafter referred to as PeptiMatrix 12.5) was centrifuged to remove air bubbles (3 min at 300×*g*) and mixed with the cell pellets in medium at a dilution of 1:10 v/v. The gel was then solidified for 10 min in the humidified incubator (37 °C, 5% CO_2_) before adding the medium. The VitroGel^®^ Organoid-3 (lot 1382301, The Well Bioscience, North Brunswick, NJ, United States; purchased from Tebubio, Netherlands; hereafter referred to as VitroGel) was brought to room temperature before seeding; to create the matrix, the gel was first mixed 1:1 (v/v) with PBS and then with the cell culture medium 1:1 (v/v) in accordance with the 3D cell culture preparation guidelines for gel stabilisation of injectable hydrogels (306BR-003). The resulting hydrogel solution was used to resuspend the cell pellets for seeding. Lastly, the gels were solidified for 15 min in the humidified incubator before adding the medium. GrowDex^®^ (1.5% concentration; UPM Biomedicals, Helsinki, Finland) was brought to room temperature and diluted to 1% concentration with the culture medium using low-adhesion pipette tips. The 1% gel mix was used to directly resuspend the cell pellets for plating, and the medium was immediately added to the wells after seeding to allow gel solidification. PuraMatrix™ peptide hydrogel (Corning Inc, New York, NY, United States; cat. no. 354250) was used at a final concentration of 0.5% according to the manufacturer’s recommendations. First, the cell pellets were resuspended in a sterile sucrose solution containing 10% sucrose (Sigma-Aldrich) and 90% water to protect the cells from osmotic stress due to the initial low pH (between 2 and 4) of the hydrogel. The medium was immediately added and exchanged three times every 30 min for gelation and pH neutralisation. The cell viability and proliferation were assayed with the WST-1 assay kit from Roche (Mannheim, Germany), and gels in wells without cells served as the background correction. After 24 h and 7 days, the culture medium was replaced with a reagent-containing medium (1:10 dilution) for 2 h (37 °C, 5% CO_2_). Then, 100 μL of the reagent-containing medium was transferred to a 96-well plate and was measured for absorbance at 450 nm (SpectraMax iD3, Molecular Devices, San Jose, CA, United States). The remaining reagent-containing medium was carefully aspirated and replaced with fresh culture medium.

### 2.5 Cell and gel preparations for seeding in 96-well plates and the Mimetas MPS device

HepaRG cells were suspended in the hydrogels for plating to compare their morphologies and baseline functions under static and dynamic conditions. To standardise the conditions, the same number of cells (25,000 cells/µL) and volume (2 µL) of cell-gel suspension were used in both the static and dynamic conditions. Briefly, for seeding in different hydrogel matrices, undifferentiated HepaRG cells were collected from the flask, counted, and aliquoted for the different preparations. The cells were pelleted (100×*g* for 5 min) immediately before resuspension in freshly prepared hydrogels, as described below. As the reference hydrogel, type Ⅰ collagen from rat tail (Cultrex, lot 1648863, cat. no. 3447-020-01, R&D Systems, Minneapolis, MN, United States) was mixed 1:1 with Matrigel (growth factor reduced and phenol-red free, lot 1364001; Corning, Bedford, MA, United States) to adjust the polymerisation. First, collagen was prepared by mixing HEPES (1 M, Thermo Fisher Scientific, cat. no. 15630056) and sodium bicarbonate solution (Sigma-Aldrich) with stock collagen (5 mg/mL) in the ratio of 1:1:8 on ice. Next, Matrigel was added 1:1 to the collagen (4 mg/mL) mix. The Matrigel–collagen mix was first used for plating the cell-free blank conditions and then to resuspend the cell pellets. After plating, the gel was solidified for 15 min in the humidified incubator before adding the medium.

Because PeptiMatrix 12.5 uptake is not possible via capillary force in the ECM channel of the OrganoPlate, we worked with lower concentrations of PeptiMatrix (5 and 7.5 mg/mL, which are hereafter referred to as PeptiMatrix 5 and 7.5, respectively). Similar to PeptiMatrix 12.5 used for pre-screening, the hydrogels were first transferred to an Eppendorf tube for centrifugation to remove air bubbles (3 min at 300×*g*). For the cell-free blank conditions on-chip and on-plate, the gel was mixed at a dilution of 1:5 v/v with the medium, while the cell pellets were first resuspended in 20 µL of the medium and then mixed with 60 µL of PeptiMatrix (5 and 7.5). The gel was then solidified for 10 min in the humidified incubator (37 °C, 5% CO_2_) before adding the medium. The VitroGel Organoid-3 and GrowDex gels were prepared as described above. For the gel-free conditions, the cell pellets were mixed with the medium instead of the hydrogel.

For the on-chip seeding, 50 µL of Hank’s balanced salt solution (HBSS; Gibco, Thermo Fisher Scientific) was dispensed into the observation window to prevent evaporation and enhance optical clarity. After each gel solidification, the gels on the 96-well plate were covered with 200 µL of the medium, and all inlets and outlets of the chip were filled with 50 µL of the culture medium. The OrganoPlate was maintained static for 4 h after addition of the medium to allow further gel stabilisation. The OrganoPlate was placed on the OrganoFlow rocker platform at an angle of 7° for 8 min in a humidified incubator (37 °C, 5% CO_2_). The HepaRG cells on the 96-well plate were differentiated with increasing concentrations of DMSO over 14 days, whereas the on-chip HepaRG culture was differentiated *in situ* under flow without the addition of DMSO. The medium was changed according to the schematic shown in Figure 1.

### 2.6 On-chip immunofluorescent staining

To localise the cells in the different hydrogels on-chip, the HepaRG cells were fixed for staining using 3.7% formaldehyde (Sigma-Aldrich) in HBSS for 15 min about 24 h after seeding, followed by washing twice for 5 min each with PBS. The on-chip tissue was permeabilised with 0.3% Triton X-100 (Millipore) in PBS. After washing once with 4% FBS (Sigma-Aldrich) in PBS for 5 min, the on-chip tissues were incubated with a blocking buffer containing 2% FBS and 2% BSA (Sigma-Aldrich) with 0.1% Tween20 (Sigma-Aldrich) in PBS for 45 min. After blocking, the cells were incubated for 20 h with primary antibody solutions for cytokeratin 18 (1:50, CK 18, hepatocyte-like cell marker) and cytokeratin 19 (1:500, CK 19, cholangiocyte-like cell marker). After incubation, the stained chips were washed thrice with 4% FBS in PBS and incubated for 2 h in the dark with the secondary antibodies Alexa Fluor 594 for CK 18 (goat anti-rabbit, 1:500, Abcam, Cambridge, United Kingdom) and Alexa Fluor 488 (donkey anti-mouse, 1:500, Abcam) for CK 19. All steps were performed at room temperature and under the presence of flow. Finally, images were captured using a confocal microscope (multiphoton microscope with fluorescent lifetime imaging; Leica SP8, Wetzlar, Germany).

### 2.7 Cell viability and proliferation assessments

WST-8 (Roche, Mannheim, Germany) was used to assess the cell viability and proliferation by measuring the reduction of the tetrazolium salt to a water-soluble formazan product via mitochondrial dehydrogenase activity, which was quantified colorimetrically. The amount of formazan produced is directly proportional to the number of viable cells, which is monitored through absorbance increase. Briefly, at specified times (Figure 1B) during the experiment, the culture medium was replaced with the reagent-containing medium (1:11 dilution) for 2 h (37 °C, 5% CO_2_). After incubation, the absorbance was measured directly on-chip at 450 nm using a spectrophotometric plate reader (SpectraMax iD3). For the 96-well plate culture, 100 µL of each sample was transferred to a new 96-well plate for measurement. For the on-chip viability calculations, the inlets and outlets of the perfusion channels were combined, and the background was subtracted using the cell-free chips. Similarly, the background measurements of the corresponding cell-free wells were subtracted from the cell wells. These analyses were performed in Microsoft Excel (Redmond, WA, United States).

### 2.8 Basal secretome monitoring in the culture medium

To monitor the functional phenotype over the course of the culture, the media were pooled on the 96-well plates and the entire chip (perfusion and gel inlets and outlets) to a total of six chips/wells for each gel condition to obtain sufficient volumes for the various assays. After collection, the samples were frozen at −80 °C until further preparations for the albumin, LDH, and bile acid assays.

#### 2.8.1 Albumin

The amount of albumin secreted was determined using the DUO ELISA kit (R&D Systems, Abingdon, United Kingdom). Next, microtiter immune plates (96 well; Thermo Fisher Scientific) were coated with 2 µg/mL of the capture antibody (R&D Systems, lot CFRU0122061) in a coating solution (0.1 M of NaHCO_3_ in MilliQ) and incubated overnight at room temperature to ensure immobilisation. After coating, the plates were washed three times with a washing buffer (0.005% Tween20 in PBS) and blocked with a blocking buffer (3% BSA in PBS) for 1 h to minimise non-specific binding. A series of human albumin standard solutions (concentration range: 2.5–160 ng/mL; R&D Systems, lot P30971) was prepared in a reagent diluent (1% BSA in PBS) to create the standard curve. After blocking, the plates were washed three times and undiluted medium samples, blanks, and standards were added to the wells; the plates were then incubated at room temperature for 2 h to allow binding of albumin to the immobilised antibody. Following incubation, the plates were washed thrice with the washing buffer to remove unbound substances. The detection antibody specific to albumin (125 ng/mL; R&D Systems, lot CFWX0222061) was mixed with the reagent diluent and added to each well, and the plates were incubated at room temperature for 2 h to facilitate binding. After washing, the wells were treated with 200-fold-diluted streptavidin-horseradish peroxidase (HRP; R&D Systems, lot P338618) and incubated with light shielding at room temperature for 20 min. After washing again, the substrate solution (TMB peroxidase, substrate A and B, 9:1) was added to each well, and the reaction was allowed to develop for 20 min in the dark. The reaction was stopped by adding 1 M of H_2_SO_4_, and the absorbances were measured at 450 nm and 570 nm using a microplate reader. For analysis, the background acquired at 570 nm was subtracted from the absorbance at 450 nm (SpectraMax iD3) for all wells, and a 4-parameter logistic curve regression was performed using the standard curve.

#### 2.8.2 LDH activity

To gain insights into the damaged and dying cells, the LDH release was measured via an in-house recipe. Briefly, the reaction mixture contains TRIS buffer (Tris-HCL T-3253 with Tris-base T-4661 in water, Sigma-Aldrich), lithium L-lactate (Sigma-Aldrich, L2250), Β-nicotinamide adenine dinucleotide sodium salt (Sigma-Aldrich, N-0632), iodonitrotetrazolium chloride (Sigma-Aldrich, I-8377), and phenazine methosulfate (Sigma-Aldrich, P-9625). Then, 50 μL of the supernatant sample was pipetted into a 96-well plate and 150 µL of the reaction mixture was added to each well. After 10 min, 50 µL of the stop solution (1 M of H_2_SO_4_) was added, and the absorbances were obtained at 490 nm and 680 nm. For analysis, the absorbance acquired at 680 nm was subtracted from that obtained at 490 nm from the corresponding samples to account for plastic impurities and the blank (no cell chip/well).

#### 2.8.3 Bile acid synthesis and profiling by liquid chromatography-tandem mass spectrometry (LC-MS/MS)

The untreated and treated pooled medium samples were used to identify and quantify the bile acids using triple quadrupole LC-MS/MS (LCMS-8050, Shimadzu Corporation, Japan) via the method described in de Bruijn et al. (2022). Briefly, for the analysis, the samples were 10× concentrated by lyophilising in a Christ Alpha 1-2 LD Plus freeze-dryer and redissolved in methanol for direct measurements. The LC-MS/MS method allowed us to measure 18 bile acids, namely LCA, UDCA, HDCA, CDCA, DCA, HCA, CA, GLCA, GUDCA, GDCA, GCDCA, GCA, TUDCA, THDCA, TCDCA, TDCA, TCA, and TLCA (see Supplementary Table S1 for the mass spectrometry parameters and limit of detection (LOD) and Supplementary Figure S3 for the chromatograms). The bile acids in the samples and standards (in MeOH) were separated on a Kinetex C18 column (1.7 μm × 100 A × 50 mm × 2.1 mm, Phenomenex 00B-4475-A) along with the corresponding 2.1 mm security guard precolumn (Phenomenex AJ0-8782) using an ultrahigh-performance liquid chromatography system with gradient elution by using MilliQ water with 0.01% formic acid and methanol/acetonitrile (50:50, v/v) as mobile phases A and B, respectively. The LODs for the 18 detectable bile acids were determined as the lowest measurable concentrations with signal-to-noise ratios exceeding 5; the limit of quantification (LOQ) was set at the lowest measurable concentration with a signal-to-noise ratio exceeding 10 (see Supplementary Table S1 and chromatograms in Supplementary Figure S3). The data were collected and processed using LabSolutions software (Shimadzu). To correct for bile acid residues from the serum supplements in the culture medium (see Supplementary Figure S4), the pooled liquid from the cell-free chips/wells was measured.

### 2.9 Cytochrome P450 enzyme induction and activity

After 7 days under flow and 14 days of static culture, both the basal and chemically induced CYP enzyme activities were determined with the P450-Glo™ Assay (Promega, Madison, WI, United States) following manufacturer recommendations for non-lytic cell-based assays. Briefly, the cells were treated daily with 25 µM of rifampicin (25 mM stock in DMSO) at the indicated time points for 48 h to induce CYP 3A4 enzymatic activity; the culture medium with 0.1% DMSO served as the vehicle control. Before the daily treatment (Figure 1B), medium samples were collected for secretome analysis and all on-chip inlets and outlets were filled with 50 µL of the exposure medium (rifampicin or vehicle). After treatment, the medium was replaced with fresh HepaRG medium containing luciferin-IPA substrate (3 μM, V9001) for 45 min. In the dynamic condition, a total of 100 μL diluted luminogenic substrate was added per chip to the top and bottom channel inlets and outlets and in static condition to a total of 200 μL was added per well and 25 µL on-chip, respectively. The substrate was incubated on the rocker inside the humidified incubator (37 °C, 5% CO_2_). After incubation on-chip, we pooled the perfusion channel medium of each chip for homogenisation and consequently transferred 25 µL of this sample to a 96-well white luminometer plate (Greiner Bio-One B.V., Germany) for measurement. To initiate the luminescent reaction, 25 µL of luciferin detection agent was added for 20 min at room temperature, and the plate reading was performed using the Promega Glo Max luminometer (Madison, WI, United States) at an integration time of 0.3 s/well. The net signal was then calculated by subtracting the background luminescence value of the no-cell chip/well from the chemically treated and untreated (vehicle control) values.

### 2.10 Gene expression

The cells were lysed for each hydrogel, and the lysis products were combined to isolate sufficient RNA for real-time qPCR (RT-qPCR). For lysis, all the liquid was aspirated from each well of a chip, and a total of 50 µL of QIAzol Reagent (Qiagen GmBH, Hilden, Germany) was added. To facilitate the lysis on-chip, a gradient flow was induced from the top perfusion inlet to the bottom outlet channels. RNA isolation was then conducted using the RNeasy Mini Kit (Qiagen) according to manufacturer instructions and quality checked with the NanoDrop One microvolume UV-Vis spectrophotometer (Thermo Fisher Scientific). Only samples with RNA concentrations above 90 ng/µL, A260/280 between 1.8 and 2.1, and A260/230 above 1.5 were included in further analyses. Next, cDNA synthesis was performed using the Qiagen QuantiTect reverse transcription kit following manufacturer protocols. The RT-qPCR analysis was conducted using the SYBR method (Bio-Rad, Veenendaal, Netherlands) with validated primers (see Supplementary Table S2). Only primers with Cq values < 30 and efficiency between 90% and 110% were included to assess the relative gene expression levels. Normalisation was performed using the geometric mean of the housekeeping genes, namely, TATA-binding protein (TBP), succinate dehydrogenase complex flavoprotein subunit A (SDHA), and ubiquitin C (UBC). The relative mRNA levels were analysed in terms of the ΔCq values, with the average fold change and geometric mean of the corresponding Matrigel-collagen culture set to unity.

### 2.11 Statistical analysis

The data are presented as mean ± standard deviation. The static and dynamic experiments were independently repeated three times with six wells/chips per hydrogel. For the albumin, LDH, bile acid, and gene expression analyses, the samples were pooled from all six wells/chips to generate one replicate (n). For the viability and CYP3A4 activity evaluations, n samples represent one plate, and three technical replicates were generated per condition. The exact sample sizes (n) are specified in each of the figure legends. The statistical analyses were conducted using Prism 10.0 (GraphPad, San Diego, CA, United States). The treatment effects were assessed using unpaired multiple t-tests with Welch’s correction (marked with *), and the differences across time points or hydrogels were analysed using ordinary one-way ANOVA with Tukey’s or Dunnett’s multiple comparisons and Brown–Forsythe tests (day-to-day marked with *; gel-to-gel marked with #). The statistical significance was defined as *p* < 0.05.

## 3 Results

### 3.1 Commercially available hydrogels

Table 1 provides an overview of the animal-free hydrogels currently available in the market. These hydrogels can be categorised into two major groups, as peptides and polysaccharides, which are either synthetic or naturally derived. Despite the variations in the major component sources and chemical properties, all manufacturers report optical transparency for microscopy, low chemical-binding properties, and tunability options (e.g. stiffness, viscoelasticity, and growth factors) based on needs. Notably, all animal-free hydrogels can be used at room temperature and are either ready-to-use or undergo rapid gelation with a crosslinker or upon contact with PBS/culture medium (e.g. PeptiMatrix and VitroGel) that also neutralises pH. Once neutralised, these hydrogels are fit-for-purpose to culture different cell types and models, such as induced pluripotent stem cell (iPSC) growth niches or tumour (interaction) models (Mendonca et al., 2023; Feodoroff et al., 2023).

**TABLE 1:** List of commercially available and tested hydrogels with their major components and origin, manufacturer/supplier, physiochemical properties, and characteristics according to the manufacturer/supplier, published cell-based applications, and technical considerations.

Major component	Commercial name	Manufacturer/Supplier	Physiochemical properties and characteristics	Applied cell/tissue systems	Technical considerations
Collagen (animal-derived)	Cultrex: Type 1 collagen from rat tail	R&D Systems	• Consists of three α-chains that can combine to form a rope-like triple helix to provide tensile strength • The α-chains contain GXY repeats. Glycine is a small amino acid that fits well in the triple helix; X and Y are typically proline and hydroxyproline, which are critical for collagen stability	One of the popular options, with over 25,700 citations on PubMed dating back to 1987 with all culturable cells	Type 1 collagen from rat tail serves as reference for a 1:1 (v/v) Matrigel mixture to optimise gel polymerisation
Laminin (animal-derived)	Matrigel (reference material)	Corning Inc.	• Tumour-derived • Contains 30% collagen ⅠⅤ, 8% entactin, perlecan, transforming growth factor, epidermal growth factor, insulin-like growth factor, tissue plasminogen activator, and residual matrix metalloproteinases • 8–21 mg of protein/mL	One of the popular options, with over 14,200 citations on PubMed dating back to 1980 with all culturable cells	To optimise gel polymerisation, Matrigel is mixed with type Ⅰ collagen from rat tail (Cultrex)
Natural polysaccharide (wood-derived nanofibrillar cellulose)	GrowDex	UPM Biomedicals	• Semi-solid (from 0–120 °C) and highly viscous hydrogel • Shear-thinning material • Neutral cellulose fibre charge • Opaque visual appearance	Iron-mediated cancer cell growth with HepG2 (Phiwchai et al., 2021) Multiplex analysis of 3D cultured HepaRG (Lampe et al., 2024) Comparison of Matrigel and GrowDex with HepG2 and ovarian cancer plasmacytoid dendritic cells for testing drug sensitivity and resistance (Feodoroff et al., 2023)	Was not taken up by the extracellular matrix (ECM) channel of OrganoPlate as the capillary force did not allow uptake. GrowDex was included in static experiments
Synthetic peptide	PeptiMatrix Core	PeptiMatrix	• Self-assembling octapeptide hydrogel ready for use in different concentrations	Pre-clinical evaluation of repurposed FDA-approved drugs for acute myeloid leukaemia (James et al., 2023) Investigation of cell–ECM interactions in health and disease using human-derived MCF-7 and MDA-MB-231 breast cancer cells (Mendonca et al., 2023)	On-chip seeding required the lowest concentration for uptake via capillary force in the ECM channel, leading to exclusion of gels with higher concentrations
	PuraMatrix	Corning Inc.	• Consists of 16 amino acids formed by repeated sequences of arginine-alanine-aspartic acid-alanine, also described as RADA (1% w/v), and 99% water • Self-assembling to a 7–10-nm-diameter fibrous structure with pore sizes ranging from 50 to 400 nm • Starting pH of 2–2.5	Evaluation of chondrogenic differentiation using human adipose-derived stem cells (Nogoceke et al., 2023) *In vitro* epithelial ovarian cancer model with SKOV-3 ovarian ascites adenocarcinoma cells and human foetal normal lung fibroblast cells (Winter et al., 2021) Reprogramming of human urinal cells into induced pluripotent stem cells to homogenous population (Sun et al., 2020)	Culture medium had to be changed after seeding three times every 30 min for gelation and pH neutralisation. The gel was consequently excluded to reduce disturbance of other hydrogels
Synthetic polysaccharide	VitroGel Organoid-3	The Well Bioscience (distributed by Tebubio)	• Neutral pH • Transparent, permeable, and compatible with different imaging systems	Intestinal model for drug transport studies using Caco-2 HT29-MTX with EA.hy926 endothelial cells (Zeiringer et al., 2023) Investigation of immune cell–epithelial interactions in a tissue chip co-culture model of human gastric organoids and dendritic cells (Cherne et al., 2021) Functional baseline evaluation of a HepG2 culture (Wang et al., 2022)	Easily supports a viable culture and capillary force uptake in the ECM channel

### 3.2 Pre-screening for viability support and MPS suitability

**FIGURE 2:**
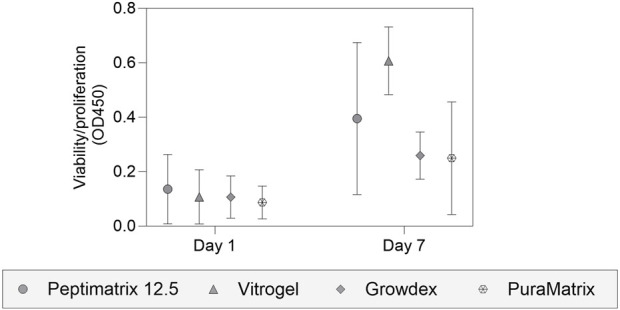
Analysis of cell viability and proliferation using the WST-1 assay. HepaRG cells were seeded in different hydrogel suspensions on 48-well plates for 7-day biocompatibility studies, including PeptiMatrix 12.5 (PeptiMatrix), VitroGel Organoid-3 (The Well Bioscience), GrowDex (UPM Biomedicals), and PuraMatrix (Corning). The data points are the mean ± standard deviation values (n = 3). Statistical differences were determined using the paired t-test and Wilcoxon matched-pairs signed rank test.

To identify biocompatible hydrogels, we pre-screened at least one hydrogel per main component category for viability support (Figure 2), namely PeptiMatrix and PuraMatrix as synthetic peptides, VitroGel as a synthetic polysaccharide, and GrowDex as a natural polysaccharide. Our pre-screening results demonstrate that all hydrogels are biocompatible with viable and growing cultures over 7 days, with differences in the capacities. VitroGel exhibited the highest proliferation support, while PeptiMatrix, GrowDex, and PuraMatrix performed similarly at a lower capacity. Subsequently, we tested the ability of the hydrogels to be absorbed into the OrganoPlate ECM channel via capillary force in preparation for the dynamic experiments. VitroGel was efficiently absorbed into the chip channel, making it a suitable candidate without any modifications. In contrast, PeptiMatrix 12.5 showed no uptake by the channel, leading us to select the lower concentrations of PeptiMatrix 5 and 7.5 with less viscosity. Similarly, GrowDex was not absorbed by the ECM channel even at the lowest working concentration recommended by the manufacturer (1%) owing to its high viscosity. PuraMatrix was ultimately excluded owing to practicality concerns as it required multiple washing steps post-seeding for pH neutralisation. Based on these short-term experiments, we selected PeptiMatrix, VitroGel, and GrowDex for further basal functional tests.

### 3.3 Cell morphology and on-chip distribution

**FIGURE 3:**
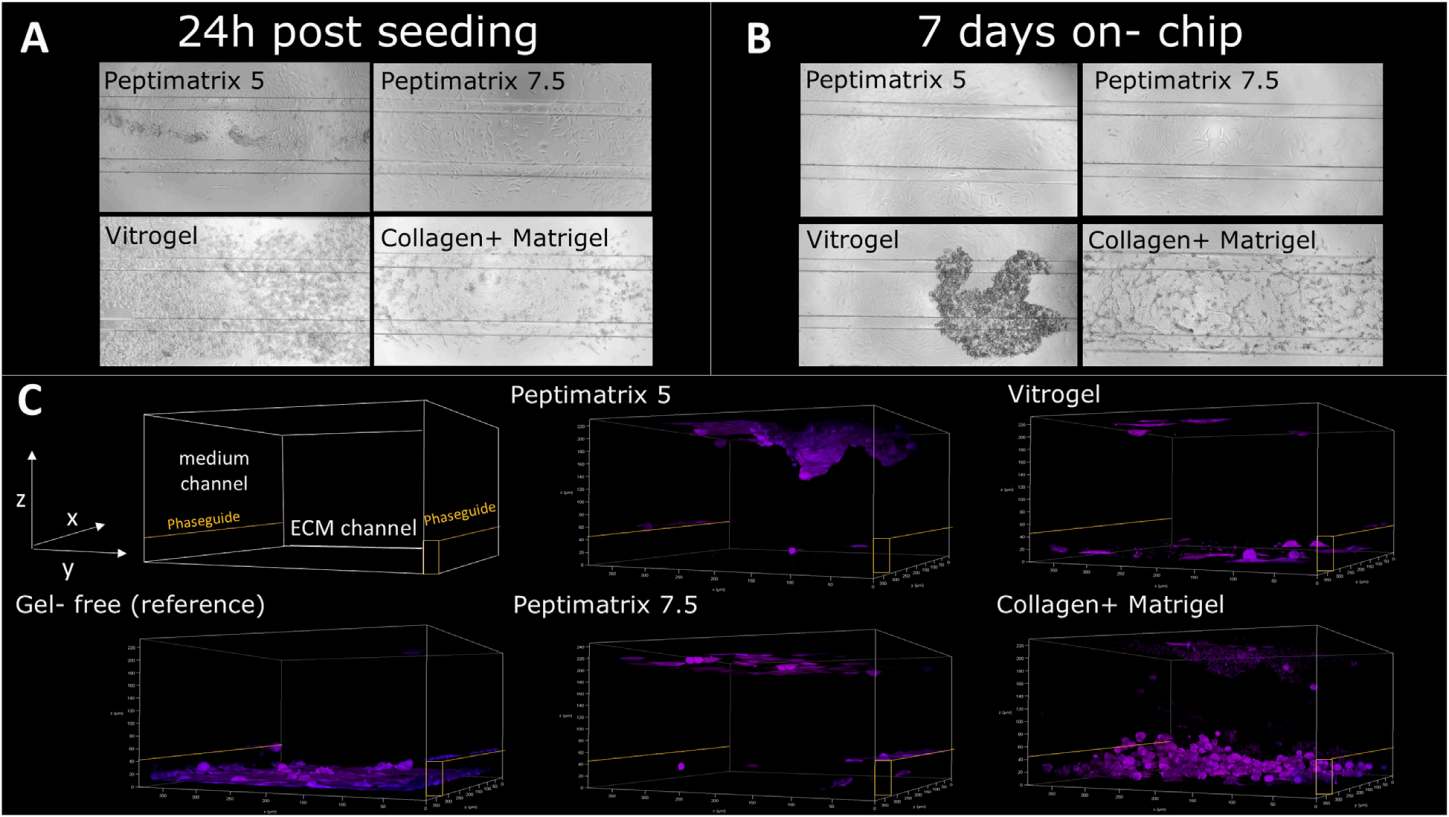
Analysis of HepaRG cell distributions and populations in PeptiMatrix 5 and 7.5 (PeptiMatrix), VitroGel Organoid-3 (The Well Bioscience), and Matrigel–collagen mix (Corning Inc. and R&D Systems) on OrganoPlate 3-lane (n = 3 plates). **(A)** Representative bright-field microscopic images (×10 magnification) of the HepaRG cells 24 h post-seeding, showing the on-chip cell distribution. **(B)** Representative bright-field microscopic images (×10 magnification) of HepaRG cells 7 days post-seeding, illustrating differences in the cell distributions and attachments. **(C)** Confocal microscopy images 24 h post-seeding with immunostaining for hepatocyte-like cells (cytokeratin 18, purple) and cholangiocyte-like cells (cytokeratin 19, green). PhaseGuides (orange) highlight the separation between the ECM and medium channels, indicating the on-chip cell localisations.

Our seeding protocol was based on the original work by Jang et al. (2019), where HepaRG cells were seeded in gel suspension on a 3-lane OrganoPlate for *in situ* differentiation without DMSO supplementation (see Figure 1B for experimental timeline). Bright-field images (Figure 3A) show that all tested hydrogels facilitated cell distribution and migration across the entire chip within 24 h post-seeding, including the medium channels, consistent with the findings reported by Jang et al. (2019). However, VitroGel demonstrated a high abundance of curled detached cells, leading to fewer attached cells after 7 days on-chip compared to the other hydrogels (Figure 3B). In contrast to the animal-free hydrogels, cells cultured in the Matrigel-collagen mix formed more complex cell structures, while the alternatives provided an attachment coating (Figure 3C). Cell accumulations were most pronounced at the top and bottom channels of the chip and near the PhaseGuides (Figure 3C), which acted as barriers to the nutrient-rich medium while offering protection from the shear flow at the bottom. Notably, immunostaining demonstrated the presence of only CK18, indicating maturation toward hepatocyte-like and absence of cholangiocyte-like cells for all tested hydrogels 24 h post-seeding (Figure 3C). This contrasts the findings of Jang et al. (2019), who reported that Matrigel promotes differentiation towards cholangiocyte-like cells.

### 3.4 Cell health and basal liver function under static conditions

**FIGURE 4:**
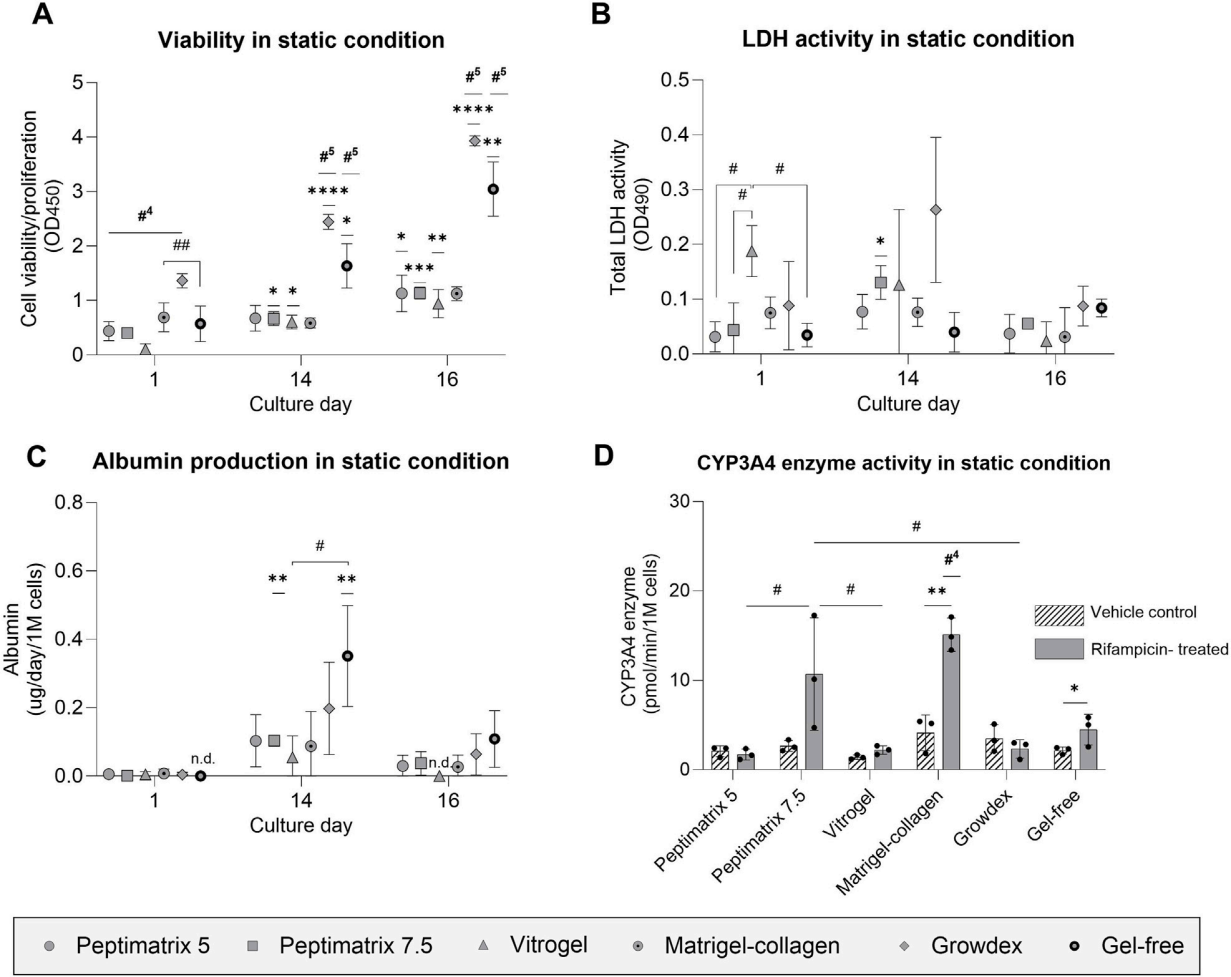
Analysis of cell health and synthetic capacity under the static condition. **(A)** Cell viability/proliferation (n = 3); **(B)** cellular membrane damage (n = 3); **(C)** albumin secretion (n = 3) normalised by the seeding cell count; **(D)** CYP3A4 enzyme activity under treatment with either vehicle control (0.1% DMSO) or rifampicin (25 µM) (n = 3) normalised by the seeding cell count. **(A–C)** The data are presented as mean ± SD and normalised by wells containing only gel without cells. Statistical differences with respect to the first day were determined by ordinary one-way ANOVA with Dunnett’s multiple comparison and Brown–Forsythe tests (*p < 0.05, **p < 0.01, ***p < 0.001, ****p < 0.0001). Statistical differences with respect to the other gels were determined by ordinary one-way ANOVA with Dunnett’s multiple comparison and Brown–Forsythe tests (^#^ *p* < 0.05, ^##^ *p* < 0.01, ^###^ *p* < 0.001); #^4^ indicates statistical difference with the four other gel cultures, while #^5^ indicates statistical difference with all cultures. **(D)** Treatment effects were assessed using multiple unpaired t-tests with Welch’s correction and are denoted by *p < 0.05 and **p < 0.01. Statistical differences with respect to the other gels were determined by ordinary one-way ANOVA with Tukey’s multiple comparison and Brown–Forsythe tests (^#^ *p* < 0.05); #^4^ indicates statistical difference with all cultures except PeptiMatrix 7.5, and ‘n.d.’ indicates not detected.

In standard HepaRG culture protocols, ECM hydrogels are generally not required unless structural support is desired (Godoy et al., 2013; Ramaiahgari et al., 2017; Ramaiahgari and Ferguson, 2019). Consistently, the best cell health over time was observed in the gel-free environment and was characterised by enhanced cell viability as well as low levels of LDH release throughout the culture period compared to the other hydrogels (Figure 4A). In contrast, cells cultured in VitroGel initially displayed the lowest viability and highest LDH leakage, matching the microscopy observations of cell curling and detachment at 24 h post-seeding (Supplementary Figure S1). Remarkably, the cells cultured in VitroGel recovered and subsequently exhibited improved viability. All animal-free gels enabled proliferation of the HepaRG cells until 16 days in culture despite DMSO supplementation for differentiation induction (Figures 4A,B). Only the Matrigel–collagen culture remained unchanged in terms of viability throughout the culture period, suggesting no further proliferation but maturation into hepatocyte-like cells. Among the tested hydrogels, cells cultured in GrowDex showed the highest initial viability, which significantly exceeded those of the other hydrogels at all time points except for the gel-free culture (Figure 4A). We hypothesise that the high gel viscosity resulted in gel retention within the low-adhesion pipette tip during mixing with cells, leading to a higher initial seeding density than the other hydrogels. This discrepancy in seeding density could also explain the higher LDH leakage as a greater number of cells was present to release the enzyme (Figure 4B).

To evaluate the maturation status and synthetic capacity, albumin and CYP3A4 enzymatic activities were assessed as the key liver markers. After 14 days, albumin production significantly increased only for the gel-free and PeptiMatrix 7.5 cultures, reaching approximately 0.1 µg/d/1 M cells before declining to levels similar to those observed 24 h post-seeding (Figure 4C). The basal CYP3A4 levels did not differ significantly across the hydrogels (Figure 4D); however, treatment effects were observed only in HepaRG cells cultured in the Matrigel–collagen and gel-free conditions. The gel-free cultured cells reached induced CYP3A4 activity levels of approximately half of those observed in the Matrigel–collagen mix, whereas cells cultured in PeptiMatrix 5, VitroGel, and GrowDex showed markedly lower activities. Collectively, GrowDex and VitroGel demonstrated the greatest support for cell proliferation, whereas the gel-free and Matrigel–collagen mixtures provided the most suitable culture conditions for CYP3A4 metabolism. Nonetheless, none of the tested animal-free hydrogels supported full maturation into CYP3A4 induction-competent HepaRG cells.

### 3.5 Functional baseline under dynamic conditions

**FIGURE 5:**
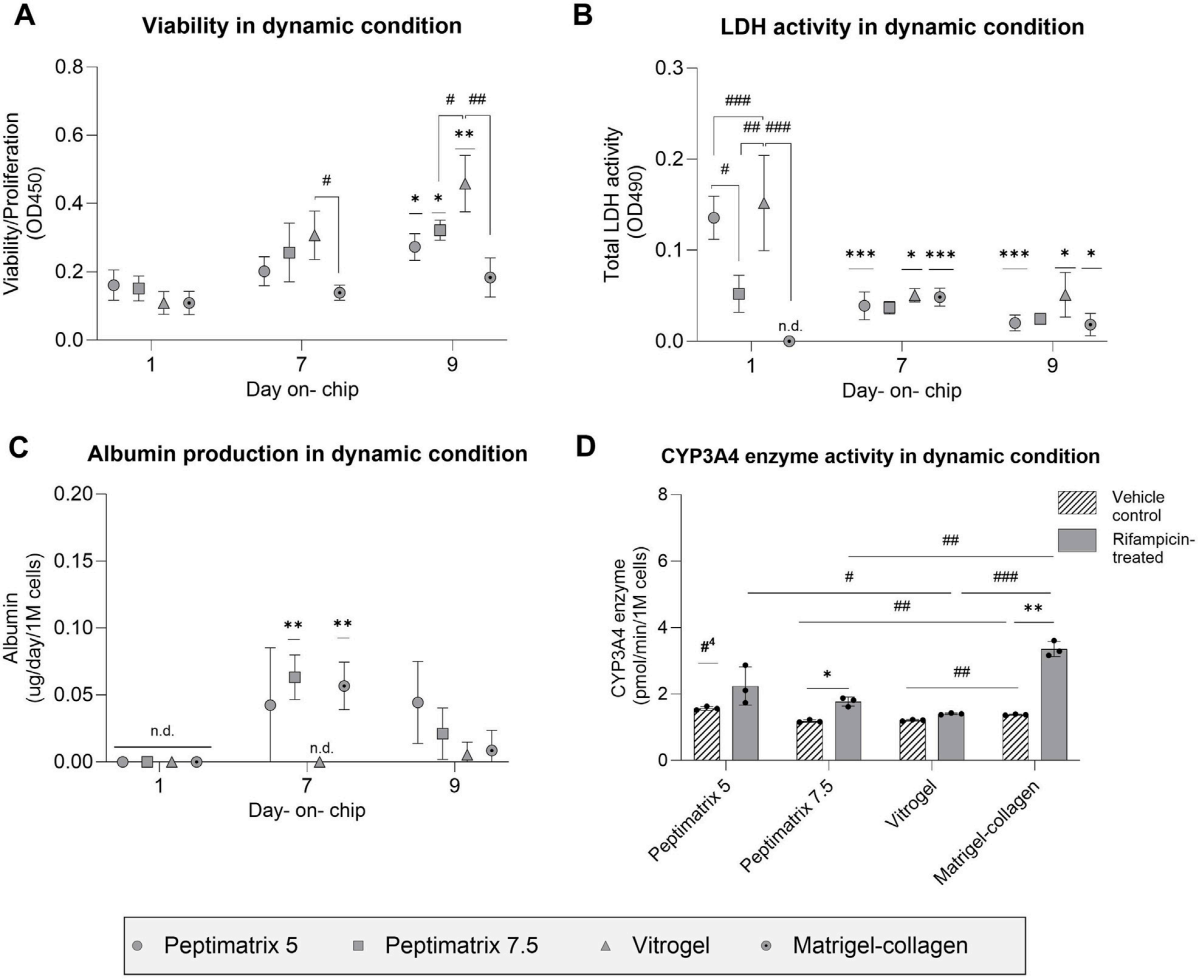
Analysis of cell health and synthetic capacity under the dynamic condition. **(A)** Cell viability/proliferation (n = 3); **(B)** cellular membrane damage (n = 3); **(C)** albumin secretion (n = 3) normalised by the seeding cell count; **(D)** CYP3A4 enzyme activity under treatment with either vehicle control (0.1% DMSO) or rifampicin (25 µM) (n = 3). **(A–C)** The data are presented as mean ± SD and normalised by chips containing only the gel without cells. Statistical differences with respect to the first day were determined by ordinary one-way ANOVA with Dunnett’s multiple comparison and Brown–Forsythe tests (*p < 0.05, **p < 0.01, ***p < 0.001). Statistical differences with respect to the other gels were determined by ordinary one-way ANOVA with Tukey’s multiple comparison and Brown–Forsythe tests (^#^ *p* < 0.05, ^##^ *p* < 0.01, ^###^ *p* < 0.001, ^####^ *p* < 0.0001). **(D)** Treatment effects were assessed using multiple unpaired t-tests with Welch’s correction and are denoted by *p < 0.05. Statistical differences with respect to the other gels were determined by ordinary one-way ANOVA with Dunnett’s multiple comparison and Brown–Forsythe tests (^#^ *p* < 0.05, ^##^ *p* < 0.01, ^###^ *p* < 0.001); #^4^ indicates statistical difference with respect to all cultures, and ‘n.d.’ indicates not detected.

Similar to the static experiments, we assessed the cell health and liver-specific functioning under the dynamic culture environment. Interestingly, all hydrogels showed comparable viabilities 24 h post-seeding, including VitroGel, despite the initial challenges with cell attachment (Figures 3B,C, 5A). Compared to day 1, the cells in VitroGel displayed the largest increase in viability, whereas the cells in both PeptiMatrix hydrogels exhibited more moderate yet significant increases over the culture period. In contrast, cells in the Matrigel–collagen mix maintained stable viability, similar to that observed under static conditions, suggesting early maturation rather than continued proliferation. Additionally, LDH leakage was not detected when the cells were cultured in the Matrigel–collagen mix, unlike the other hydrogels. This outcome may be attributed to the continuously stable viability and possibly slower biomolecule diffusion from the ECM to the medium (Kihara et al., 2013; Proença et al., 2021; Solbu et al., 2023). By days 7 and 9, the cells in PeptiMatrix 5 and VitroGel showed progressive reductions in LDH leakage, suggesting that the cells were adapting to the conditions within the chip (Figure 5B).

To assess the maturation status and on-chip synthetic capacity, albumin secretion and CYP3A4 enzymatic activities were analysed. By day 7, the cells cultured in PeptiMatrix 7.5 and the Matrigel-collagen mix not only secreted the highest levels of albumin but also showed significant treatment responses, suggesting maturation toward CYP induction-competent cells under flow conditions (Figure 5C). Interestingly, while the cells in all hydrogels exhibited lower basal CYP3A4 enzyme levels compared to those in PeptiMatrix 5, the cells in PeptiMatrix 5 did not show a measurable response to induction treatment. Contrary to the observations in all other hydrogels, the cells cultured in VitroGel initially lacked albumin secretion but showed basal CYP 3A4 levels, indicating a limited but gradually increasing synthetic function in this culture setting (Figure 5D). The treatment response observed in the Matrigel–collagen mix was expected as these cells likely benefitted from the tumour-derived bioactive proteins that enhance differentiation (Kleinman and Martin, 2005; Talbot and Caperna, 2015). Despite the absence of bioactive proteins, the cells cultured in PeptiMatrix 7.5 were able to secrete albumin, albeit to a lesser extent, possibly owing to the enhanced mechanobiology (Ohashi et al., 2017). In summary, although all tested hydrogels support proliferation, only PeptiMatrix 7.5 appears to be a promising candidate for metabolic studies under flow, while the other hydrogels require optimisation for an enhanced maturation support before being employed.

### 3.6 Primary (conjugated) bile acid secretion

**FIGURE 6:**
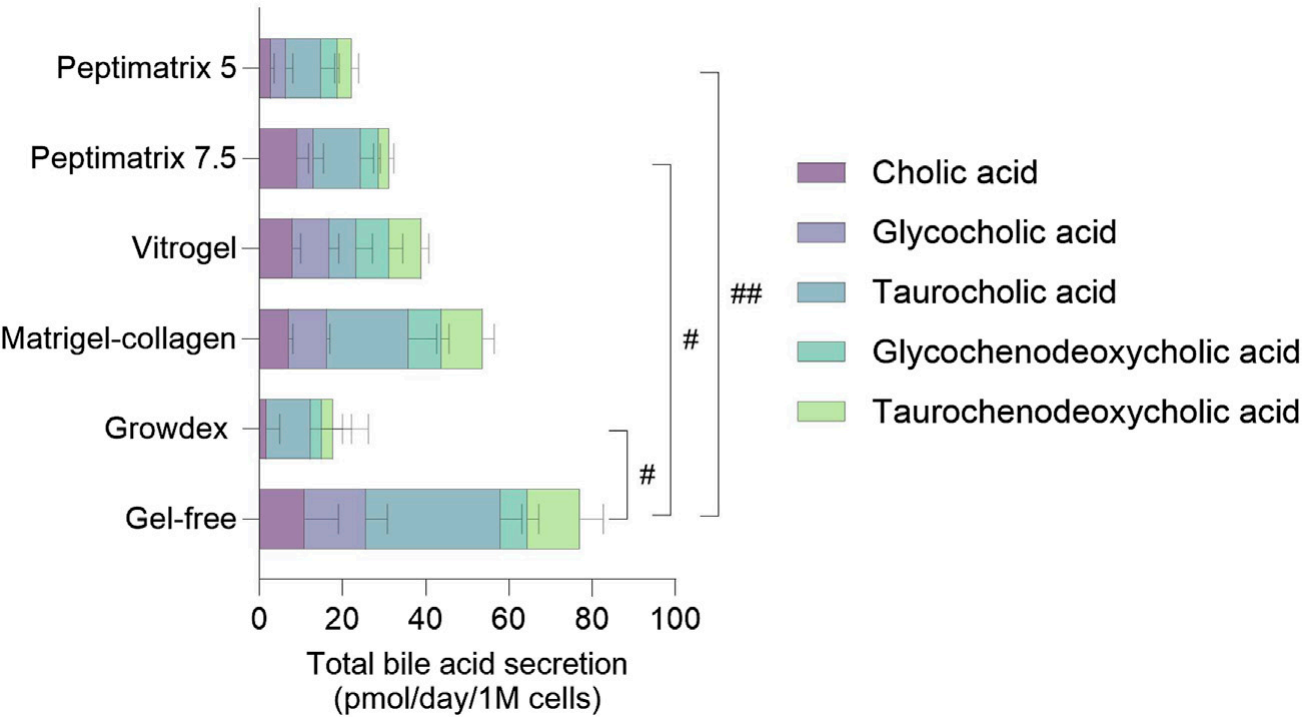
Quantification of primary (conjugated) bile acid secretion in the pooled cell-culture medium of statically cultured HepaRG on day 14. The values are presented as mean ± SD of six pooled wells normalised by the initial seeding cell count and corrected for the bile acid amounts present in the supplemented culture medium (see Supplementary Figure S4). The concentrations of the remaining 13 bile acids were below the limit of quantification (LOQ) (see Supplementary Table S1 for the limits of detection (LODs) and LOQs). Statistical differences between the hydrogels were determined by ordinary one-way ANOVA with Tukey’s multiple comparison and Brown–Forsythe tests (^#^ *p* < 0.05, ^##^ *p* < 0.01, n = 3).

Primary (conjugated) bile acid secretion was measured as an additional marker of hepatocyte synthetic functioning. Under static culture conditions, the cells in all hydrogels enabled secretion of cholic acid (CA), glycocholic acid (GCA), taurocholic acid (TCA), glycochenodeoxycholic acid (GCDCA), and taurochenodeoxycholic acid (TCDCA) (Figure 6) except for GrowDex, which lacked overall GCA secretion. While the pool compositions varied considerably in the individual bile acid abundances across hydrogels, a matrix effect was only observed in the gel-free culture and not among the tested hydrogels. Across all hydrogels, CA conjugates were secreted most abundantly, with a preference for the tauro-conjugates. The gel-free culture exhibited the highest total bile acid secretion (77 ± 5 pmol/d/1 M), whereas the cells in the alternative hydrogels secreted approximately half as much, mirroring the albumin secretion trends (Figure 4B). Under dynamic culture conditions, the cells in all hydrogels, including the Matrigel–collagen mix, exhibited even lower secretion quantities and diversity (Supplementary Figure S5). These findings suggest that while all tested hydrogels enable at least low amounts of bile acid secretions under static conditions, the dynamic culturing conditions do not sustain bile acid secretions.

### 3.7 Relative gene expression profile to corresponding Matrigel-collagen cultures

**FIGURE 7:**
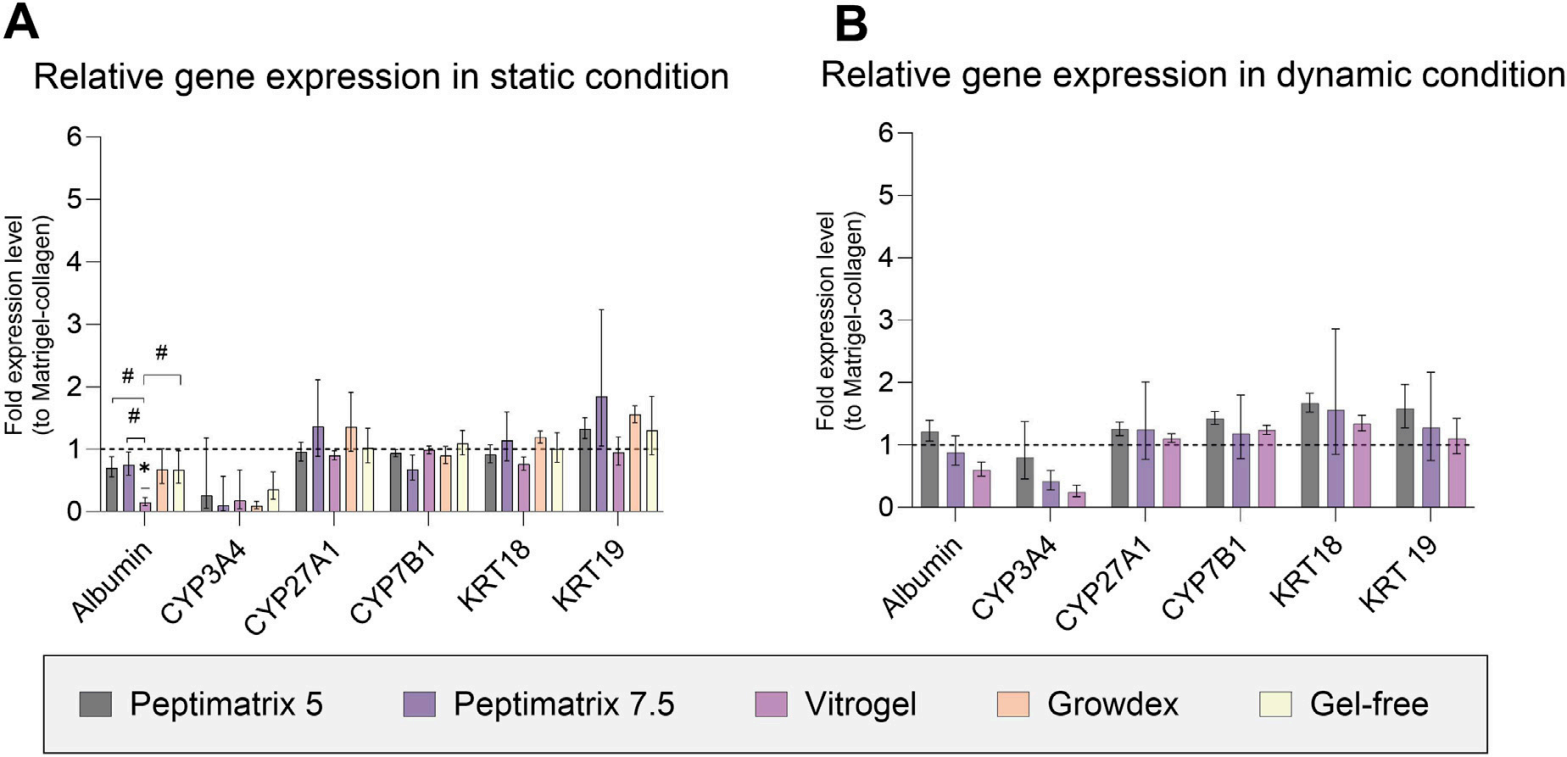
Fold changes in gene expressions for albumin; cytochrome P (CYP) 3A4, 27A1, and 7B1; and cytokeratin (CK) 18 and 19. **(A)** Relative gene expression after 16 days in static culture (n = 3; n = 2 for CYP3A4 of PeptiMatrix 5). **(B)** Relative gene expression after 9 days in dynamic culture (n = 3). The data are the geometric means with upper and lower limits from six pooled chips or wells. Statistical differences with respect to the corresponding Matrigel–collagen cultures were determined by ordinary one-way ANOVA with Dunnett’s multiple comparison and Brown–Forsythe tests (*p < 0.05). Statistical differences between the hydrogels were determined by ordinary one-way ANOVA with Tukey’s multiple comparison and Brown–Forsythe tests (^#^ *p* < 0.05).

To investigate the effects of the different seeding matrices and culture conditions on the relative gene expressions, we analysed six different genes involved in synthetic functioning (albumin, bile acid-related CYP 27A1 and CYP7B1), chemical biotransformation (CYP 3A4), and cell population (cytokeratins 18 and 19) after the final day of culture (Figure 7). The gene expression profiles were largely similar between the dynamic and static conditions as well as across different seeding matrices. However, in the static culture, cells in VitroGel exhibited reduced albumin gene expression, consistent with the absence of albumin secretion observed on the same day and in the other hydrogels (Figure 4C). Additionally, we found that the RNA extracted from HepaRG cells cultured in-gel appeared more unstable, exhibiting significant variability across the already limited pooled samples. Together, there were no significant differences in the relative gene expressions between the hydrogel and corresponding Matrigel-collagen cultures, consistent with the findings on cell health and functioning except for the differences observed in the basal CYP3A4 activities under dynamic conditions (Figure 5D).

## 4 Discussion

**FIGURE 8:**
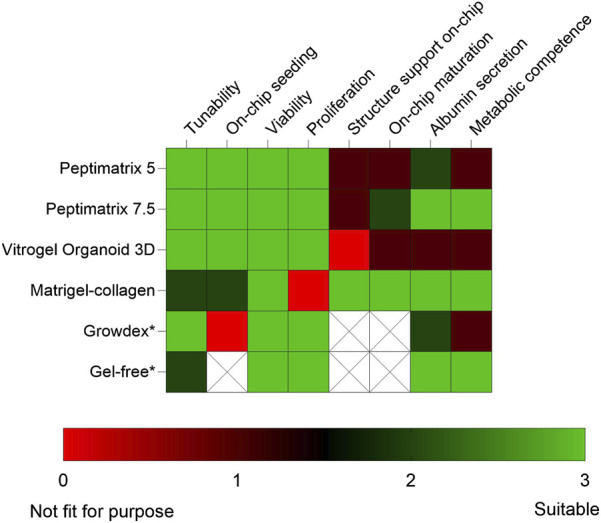
Heatmap summarising the findings and comparing the tested alternative hydrogels with the Matrigel–collagen and gel-free mixtures under static (*) and dynamic culture conditions. Colour grading was used to compare the application considerations and biocompatibility aspects for creating viable and metabolically competent HepaRG cultures. The blank regions indicate that the corresponding categories were not applicable. The factors were evaluated by (i) reviewing the characteristics described in literature, (ii) injecting hydrogels into the OrganoPlate at different concentrations, (iii) assaying for viability and proliferation, (iv) staining for cell distribution and on-chip population, as well as (v) measuring the basal functions after *in situ* differentiation under both static and dynamic conditions.

The future implementations of NAMs are aimed at animal-free chemical risk assessments. However, *in vitro* NAM models like iPSCs and 3D cultures in both conventional and MPS devices continue to rely heavily on animal-derived products like Matrigel and collagen (Punt et al., 2017; Oredsson et al., 2019). Despite the availability of alternative hydrogels, animal-based matrices remain the most popular option and have generated increasing global sales (QYResearch, 2024), even though they present ethical, reproducibility, and biomedical limitations (Oredsson et al., 2019; Aisenbrey and Murphy, 2020; Duarte et al., 2023). In this explorative study, we assessed commercially available animal-free hydrogels using a three-tiered approach. First, we conducted a literature review to characterise different properties of hydrogels and select screening candidates; then, we conducted pre-screening under static conditions for HepaRG cell biocompatibility and MPS compatibility assessment; lastly, we performed functional characterisation of HepaRG cells maturing in each hydrogel under both static and dynamic conditions. Our findings show that despite differences in the compositions, all tested animal-free hydrogels supported viable cultures and thus hold potential as substitutes depending on the application (Figure 8). Notably, CYP3A4 inducibility as a key marker of hepatic function was observed in the reference Matrigel–collagen culture under both static and dynamic conditions, gel-free culture under static conditions, and alternative PeptiMatrix 7.5 culture under dynamic conditions. These findings suggest that while not all hydrogels support maturation towards metabolically competent HepaRG cells in this culture setting, PeptiMatrix 7.5 demonstrates potential for functional liver applications after further optimisation.

This explorative study was motivated by not only the need for more animal-free NAMs but also the growing interest in MPSs, which rely heavily on ECM hydrogels for seeding to offer physiologically relevant tissue environments for the cells. The reviewed studies (Table 1) highlight the growing market for ECM alternatives, showcasing their versatility and broad applicability across various cell models for cell adhesion, proliferation, and differentiation. However, this versatility is also reflected in the diverse technical considerations for gel preparation (e.g., using medium or PBS/HBSS for dilution, washing with sucrose solution, and need for low-adhesion pipettes) and gel stabilisation (e.g., by medium/PBS addition or temperature control). Moreover, manufacturer protocols provide very little guidance for injectable hydrogels, particularly for MPS devices that depend on surface tension and capillary forces for gel uptake. Herein, we followed the manufacturer protocols but standardised certain culture parameters (e.g., equalising gel volumes for droplet seeding, using standard well plates, and using 10% FBS in medium) to allow comparisons between the static and dynamic conditions as well as findings from literature; we thus acknowledge this can be a potential confounding factor in the observed cell performances. Further, the short-term pre-screening included only four hydrogels since we focused on assessing the biocompatibility because traditional HepaRG culture models typically do not employ hydrogels unless structural support is desired in the culture configuration (Ramaiahgari et al., 2017; Ramaiahgari and Ferguson, 2019). The pre-screening results (Figure 2) confirmed that all four alternative hydrogels were biocompatible with the HepaRG cells as they supported viable cultures, albeit with differences in their ease of handling and gel uptake by the Mimetas MPS device. Based on these findings, we conducted further evaluations with the lowest concentrations of PeptiMatrix hydrogels, VitroGel Organoid-3, and GrowDex for static culturing. As an animal-derived reference gel, we used a 1:1 Matrigel–collagen mixture; this combination was selected because Matrigel alone has been shown to restrict oxygen and nutrient diffusion, potentially leading to zonation-like effects that may reduce synthetic functions (Griffith and Swartz, 2006; Colom et al., 2014; McMurtrey, 2016). The dilution with collagen was chosen to maintain the structural protein content, improve diffusion, and avoid compromised polymerisation.

When structural and biological supports are required for the cells, researchers commonly rely on animal-derived ECM hydrogels like Matrigel and collagen for coating and scaffolding, often in combination with FBS as the medium supplement. While these components provide culture support, they inherently contain bioactive molecules whose effects on the cell behaviours can be either beneficial or detrimental depending on the application (Kleinman and Martin, 2005; Talbot and Caperna, 2015; van der Valk, 2018). Under static conditions, the gel-free cell culture showed the best cell health with increasing viability, low LDH leakage, and highest albumin and bile acid synthesis, whereas cells cultured in the Matrigel–collagen mix maintained stable viability with approximately two-fold higher basal and induced CYP3A4 enzyme levels (Figures 3, 6). The differences in the enzyme levels highlight the impacts of the bioactive molecules as undifferentiated HepaRG cells in the Matrigel–collagen mix transitioned from proliferation to maturation earlier than in the alternative hydrogels. This effect may be linked to integrin-mediated binding, which provides signalling cues that drive specific biological functions (Meng et al., 2010; Kozlowski et al., 2021). However, the overall basal synthetic capacity remained low for cells cultured in gel under static conditions (Figure 4), with albumin levels below 1–5 µg/d/1 M cells for differentiated 2D HepaRG (Lübberstedt et al., 2011; Ott et al., 2017; Teng et al., 2021). We hypothesise that the differentiation protocol, particularly the requisite DMSO supplementation, contributed to albumin production suppression, as also observed by Pal et al. (2012) and Rebelo et al. (2015). Interestingly, despite some functional differences in the synthetic capacities (e.g. bile acid and albumin secretion, CYP3A4 activity), the cells cultured in gel-free and all animal-free hydrogel media showed comparable gene expressions to the Matrigel–collagen mix (Figure 7). Only the cells cultured in VitroGel displayed reduced albumin gene expression and production. Nevertheless, all hydrogels supported sustained cell proliferation over 16 days, indicating their potential utility for cell expansion, even if full hepatic functionality was not achieved under these conditions.

MPS technology is rapidly gaining popularity, so an increasing number of companies are offering diverse platform designs and applications (Wu et al., 2020; Nitsche et al., 2022; Rusyn et al., 2022). Our study utilises an MPS device with a closed culture compartment design that relies on ECM seeding, necessitating hydrogel injection for creating the environment to host cells and guide media flow (Jang et al., 2018; Bircsak et al., 2021; Mimetas, 2022; Nitsche et al., 2022). However, successful injection depends on factors like the capillary surface charge and gel viscosity. The formation of a stable and perfusable matrix requires delicate interplay between timely and appropriate matrix stabilisation and limited gel swelling (Berthier et al., 2019; Liu et al., 2019; D’Orazio et al., 2024). Our pre-screening findings indicate that only low-viscosity hydrogels successfully filled the closed culture compartment, rendering even the lowest concentration of GrowDex unsuitable for seeding cells in the OrganoPlate. Practical solutions for handling high-viscosity gels, such as using repeater pipettes, low-retention pipette tips, low-adhesion plates, and pre-warming the gels, could improve manageability, although further experiments are required to confirm whether these strategies could achieve optimal cell seeding density and gel stability. Moreover, direct comparisons between the hydrogels based on mechanobiological properties remain limited as the lack of manufacturer-provided rheological data on stiffness and viscoelasticity restrict the ability to directly correlate gel mechanics with observed cell behaviours.

Herein, only the Matrigel-collagen mix polymerised to a matrix that supported more complex on-chip cell organisation (Figure 3C). This matrix promoted self-organisation to cell clusters, likely influenced by the bioactive molecules present (Kleinman and Martin, 2005; Talbot and Caperna, 2015). The matrix also acted as a diffusion barrier, which explains the absence of detectable LDH leakage on day 1 compared to the other hydrogels (Kihara et al., 2013; Solbu et al., 2023). Although the other animal-free hydrogels failed to support more complex on-chip cell organisation, they provided attachment surfaces and enhanced proliferation over 9 days (Figure 5A). However, same as in the static condition, the overall synthetic functioning was consistently suboptimal for cells in all hydrogels. Albumin secretion remained below the reported levels of 7–15 µg/d/1 M cells for dynamically cultured HepaRG cells (Teng et al., 2021; Novac et al., 2022; Kopp et al., 2024) and the secreted bile acids exhibited reduced quantity and diversity (see Supplementary Figure S5), similar to those under static conditions (Figure 6). Among the tested animal-free hydrogels on-chip, only PeptiMatrix 7.5 supported maturation toward CYP3A4 induction-competent HepaRG cells under flow without additional growth factors, although its enzyme levels remained lower than those observed in the Matrigel-collagen culture. While these results suggest PeptiMatrix 7.5 as a promising candidate for xenobiotic metabolism studies, it is important to emphasise that CYP3A4 inducibility was not observed with cells in the other alternative hydrogels and that further culture optimisation will be required before broader applicability.

Based on our findings, we propose several optimisation steps in follow-up research. As a first step, we suggest biological tuning of the alternative hydrogels with animal-free recombinant growth factors and cytokines to create a controlled environment that supports cell maturation and synthetic functioning (Yan et al., 2018). Further adjustments to enhance the synthetic functioning may include different seeding densities (Ip et al., 2024), extended culture times for maturation, cell pre-differentiation (Hammour et al., 2022), and modifications to the shear stress conditions (Alzebdeh and Matthew, 2017). Additionally, mechanical property tuning (e.g. hydrogel stability and stiffness) could include testing of the concentration ranges offered by manufacturers, customising the crosslinker ratios in-house (e.g. culture medium and chemical modifiers), or creating hybrid hydrogels that optimise mesh size and packing density, thereby enhancing nutrient and oxygen diffusion (Li et al., 2021). Similarly, switching from suspension seeding to coating can improve the supply of nutrients and oxygen while mitigating unintended liver-like zonation effects that may reduce the synthetic capacity (van Wenum et al., 2018; Ribeiro et al., 2019). Such zonation effects or more precisely their absence may explain the superior performances observed in the static and gel-free cell cultures (e.g. albumin and bile acid secretion) as well as the improvements in cell health and synthetic functioning of cells cultured in VitroGel despite the initial cell losses. Oxygen availability is a particular limitation in closed MPSs. Lim et al. (2023) demonstrated that primary human hepatocytes underperform compared to iPSC-derived hepatocytes in the OrganoPlate owing to insufficient medium reoxygenation to meet the oxygen consumption rates of the cells (Lee-Montiel et al., 2017; Mas-Bargues et al., 2019). Consequently, we propose using cell sources with lower oxygen requirements, such as iPSC-derived hepatocytes, when employing suspension seeding or closed MPS devices or even the exploration of MPS devices with open culture compartments like the PhysioMimix (LC 12 by CN Bio, Cambridge, United Kingdom) or the HUMIMIC (TissUse GmbH, Berlin, Germany) (Tao et al., 2021; Cox et al., 2022; Novac et al., 2022). Ideally, uncoated scaffolds in open MPS devices can be coated with alternative hydrogels in-house or MPS manufacturers can take the initiative to move beyond collagen-coated scaffolds toward hydrogel alternatives, thereby contributing to true animal-derivative reduction in NAMs (Weber et al., 2022; Duarte et al., 2023; Pamies et al., 2024). To advance toward fully animal-free NAMs, future culture setups and assay protocols should also eliminate the remaining animal-derived components. In this study, 10% FBS was used for nutrient supply and experimental consistency alongside trypsin for cell collection as well as BSA and animal-derived antibodies for immunostaining. Recent promising studies have demonstrated that FBS can be replaced by chemically defined serum-free supplements without compromising cell performance, highlighting both the necessity and opportunity for further elimination of animal-derived components (Rafnsdóttir et al., 2023; Cochrane et al., 2024; Pfeifer et al., 2024; Malakpour-Permlid et al., 2025).

In conclusion, this study highlights the potential of animal-free hydrogels as alternative ECMs depending on their application (Figure 8). All tested hydrogels were biocompatible with HepaRG cells and supported their proliferation and viability, although there were limitations in the synthetic capacity likely resulting from insufficient oxygen and nutrient delivery. Notably, CYP3A4 inducibility as a key hepatic marker was observed in cells cultured in PeptiMatrix 7.5 under dynamic conditions without the added bioactive molecules, albeit at lower levels than the Matrigel–collagen reference. These findings indicate that not all alternative hydrogels currently support functional maturation of HepaRG cells in this culture setting, emphasising the need for further optimisation before they can be applied in functional liver models. Future efforts that enhance hepatic functionality with alternative hydrogels could focus on mechanobiological tuning, evaluation of iPSC-derived hepatocytes in the OrganoPlate, and exploration of alternative MPS platforms. Although MPS optimisations are limited by their low throughput, device complexity, and cost (Singh et al., 2021; Rusyn et al., 2022; Duarte et al., 2023), we encourage combined exploration with animal-free hydrogels as their broader adoption can improve human relevance, reproducibility, and standardisation of complex *in vitro* models, thereby bridging the gap between discovery and clinical research.

## Data Availability

The raw data supporting the conclusions of this article will be made available by the authors without undue reservation.

## References

[B1] Aisenbrey E. A. Murphy W. L. (2020). Synthetic alternatives to Matrigel. Nat. Rev. Mater. 5, 539–551. 10.1038/S41578-020-0199-8 32953138 PMC7500703

[B2] Alzebdeh D. A. Matthew H. W. (2017). Metabolic oscillations in co-cultures of hepatocytes and mesenchymal stem cells: effects of seeding arrangement and culture mixing. J. Cell Biochem. 118, 3003–3015. 10.1002/jcb.25962 28252220

[B3] Berthier E. Dostie A. M. Lee U. N. Berthier J. Theberge A. B. (2019). Open microfluidic capillary systems. Anal. Chem. 91, 8739–8750. 10.1021/ACS.ANALCHEM.9B01429 31260266 PMC7409765

[B4] Bircsak K. M. DeBiasio R. Miedel M. Alsebahi A. Reddinger R. Saleh A. (2021). A 3D microfluidic liver model for high throughput compound toxicity screening in the OrganoPlate®. Toxicology 450, 152667. 10.1016/j.tox.2020.152667 33359578

[B5] Caliari S. R. Burdick J. A. (2016). A practical guide to hydrogels for cell culture. Nat. Methods 13, 405–414. 10.1038/nmeth.3839 27123816 PMC5800304

[B6] Charbonier F. Indana D. Chaudhuri O. (2021). Tuning viscoelasticity in alginate hydrogels for 3D cell culture studies. Curr. Protoc. 1, e124. 10.1002/cpz1.124 34000104 PMC8171168

[B7] Cherne M. D. Sidar B. Sebrell T. A. Sanchez H. S. Heaton K. Kassama F. J. (2021). A synthetic hydrogel, VitroGel® ORGANOID-3, improves immune cell-epithelial interactions in a tissue chip co-culture model of human gastric organoids and dendritic cells. Front. Pharmacol. 12, 707891. 10.3389/fphar.2021.707891 34552484 PMC8450338

[B8] Cochrane S. Saib O. Sheffield D. (2024). Use of serum-free media for peripheral blood mononuclear cell culture and the impact on T and B cell readouts. Front. Toxicol. 6, 1462688. 10.3389/ftox.2024.1462688 39563982 PMC11573784

[B9] Colom A. Galgoczy R. Almendros I. Xaubet A. Farré R. Alcaraz J. (2014). Oxygen diffusion and consumption in extracellular matrix gels: implications for designing three-dimensional cultures. J. Biomed. Mater. Res. A 102, 2776–2784. 10.1002/JBM.A.34946 24027235

[B10] Corning Inc. (2023). Corning ® Matrigel ® matrix frequently asked questions. Available online at: https://www.corning.com/catalog/cls/documents/faqs/CLS-DL-CC-026.pdf (Accessed October 14, 2024).

[B11] Cox B. Barton P. Class R. Coxhead H. Delatour C. Gillent E. (2022). Setup of human liver-chips integrating 3D models, microwells and a standardized microfluidic platform as proof-of-concept study to support drug evaluation. Biomater. Biosyst. 7, 100054. 10.1016/J.BBIOSY.2022.100054 36824483 PMC9934436

[B12] de Bruijn V. M. P. Wang Z. Bakker W. Zheng W. Spee B. Bouwmeester H. (2022). Hepatic bile acid synthesis and secretion: comparison of *in vitro* methods. Toxicol. Lett. 365, 46–60. 10.1016/J.TOXLET.2022.06.004 35724847

[B13] Duarte A. C. Costa E. C. Filipe H. A. L. Saraiva S. M. Jacinto T. Miguel S. P. (2023). Animal-derived products in science and current alternatives. Biomater. Adv. 151, 213428. 10.1016/j.bioadv.2023.213428 37146527

[B14] Duivenvoorde L. P. M. Louisse J. Pinckaers N. E. T. Nguyen T. van der Zande M. (2021). Comparison of gene expression and biotransformation activity of HepaRG cells under static and dynamic culture conditions. Sci. Rep. 11, 10327. 10.1038/s41598-021-89710-6 33990636 PMC8121841

[B15] D’Orazio M. Filippi J. Antonelli G. Curci G. Casti P. Mencattini A. (2024). Cells in the 3D biomatrix on-chip: better mimicking the real micro-physiological system. Next Mater. 5, 100229. 10.1016/j.nxmate.2024.100229

[B16] Feodoroff M. Mikkonen P. Turunen L. Hassinen A. Paasonen L. Paavolainen L. (2023). Comparison of two supporting matrices for patient-derived cancer cells in 3D drug sensitivity and resistance testing assay (3D-DSRT). SLAS Discov. 28, 138–148. 10.1016/j.slasd.2023.03.002 36934951

[B17] Frantz C. Stewart K. M. Weaver V. M. (2010). The extracellular matrix at a glance. J. Cell Sci. 123, 4195–4200. 10.1242/jcs.023820 21123617 PMC2995612

[B18] Godoy P. Hewitt N. J. Albrecht U. Andersen M. E. Ansari N. Bhattacharya S. (2013). Recent advances in 2D and 3D *in vitro* systems using primary hepatocytes, alternative hepatocyte sources and non-parenchymal liver cells and their use in investigating mechanisms of hepatotoxicity, cell signaling and ADME. Arch. Toxicol. 87, 1315–1530. 10.1007/s00204-013-1078-5 23974980 PMC3753504

[B19] Griffith L. G. Swartz M. A. (2006). Capturing complex 3D tissue physiology *in vitro* . Nat. Rev. Mol. Cell Biol. 7 (37), 211–224. 10.1038/nrm1858 16496023

[B20] Hammour M. M. Othman A. Aspera-Werz R. Braun B. Weis-Klemm M. Wagner S. (2022). Optimisation of the HepaRG cell line model for drug toxicity studies using two different cultivation conditions: advantages and limitations. Arch. Toxicol. 96, 2511–2521. 10.1007/s00204-022-03329-8 35748891

[B21] Hickey R. J. Pelling A. E. (2019). Cellulose biomaterials for tissue engineering. Front. Bioeng. Biotechnol. 7, 45. 10.3389/fbioe.2019.00045 30968018 PMC6438900

[B83] Humayun L. Smith C. Li W. Zhang Y. Park C. Feng W. (2021). SARS-CoV-2-related vascular injury: mechanisms, imaging and models. Microphysiol. Syst. 5. 10.21037/mps-20-6 33981988 PMC8112618

[B22] Ip B. C. Madnick S. J. Zheng S. van Tongeren T. C. A. Hall S. J. Li H. (2024). Development of a human liver microphysiological coculture system for higher throughput chemical safety assessment. Toxicol. Sci. 199, 227–245. 10.1093/toxsci/kfae018 38335931 PMC11131024

[B23] James J. R. Curd J. Ashworth J. C. Abuhantash M. Grundy M. Seedhouse C. H. (2023). Hydrogel-based pre-clinical evaluation of repurposed FDA-approved drugs for AML. Int. J. Mol. Sci. 24, 4235. 10.3390/ijms24044235 36835644 PMC9966469

[B24] Jang M. Manz A. Volk T. Kleber A. (2018). Study of melatonin-mediated effects on various hepatic inflammatory responses stimulated by IL-6 in a new HepG2-on-a-chip platform. Biomed. Microdevices 20, 54–12. 10.1007/s10544-018-0300-x 29946898

[B25] Jang M. Kleber A. Ruckelshausen T. Betzholz R. Manz A. (2019). Differentiation of the human liver progenitor cell line (HepaRG) on a microfluidic‐based biochip. J. Tissue Eng. Regen. Med. 13, 482–494. 10.1002/term.2802 30746894

[B26] Jensen C. Teng Y. (2020). Is it time to start transitioning from 2D to 3D cell culture? Front. Mol. Biosci. 7, 33. 10.3389/fmolb.2020.00033 32211418 PMC7067892

[B27] Kihara T. Ito J. Miyake J. (2013). Measurement of biomolecular diffusion in extracellular matrix condensed by fibroblasts using fluorescence correlation spectroscopy. PLoS One 8, e82382. 10.1371/journal.pone.0082382 24312418 PMC3842966

[B28] Kleinman H. K. Martin G. R. (2005). Matrigel: basement membrane matrix with biological activity. Semin. Cancer Biol. 15, 378–386. 10.1016/j.semcancer.2005.05.004 15975825

[B29] Kopp B. Khawam A. Di Perna K. Lenart D. Vinette M. Silva R. (2024). Liver-on-chip model and application in predictive genotoxicity and mutagenicity of drugs. Mutat. Res. Genet. Toxicol. Environ. Mutagen. 896, 503762. 10.1016/j.mrgentox.2024.503762 38821675

[B30] Kozlowski M. T. Crook C. J. Ku H. T. (2021). Towards organoid culture without Matrigel. Commun. Biol. 4 (1), 1387–15. 10.1038/s42003-021-02910-8 34893703 PMC8664924

[B31] Kular J. K. Basu S. Sharma R. I. (2014). The extracellular matrix: structure, composition, age-related differences, tools for analysis and applications for tissue engineering. J. Tissue Eng. 5, 2041731414557112. 10.1177/2041731414557112 25610589 PMC4883592

[B32] Lampe J. Sheard J. Heywood D. Ahokas K. Mikkonen P. (2024). Multiplex analysis of 3D liver cell cultures in GrowDex®. Available online at: https://www.upmbiomedicals.com/resource-center/application-notes/multiplex-analysis-of-3d-liver-cell-cultures-in-growdex/(Accessed September 20, 2024).

[B33] Lee-Montiel F. T. George S. M. Gough A. H. Sharma A. D. Wu J. DeBiasio R. (2017). Control of oxygen tension recapitulates zone-specific functions in human liver microphysiology systems. Exp. Biol. Med. 242, 1617–1632. 10.1177/1535370217703978 28409533 PMC5661766

[B34] Li X. Sun Q. Li Q. Kawazoe N. Chen G. (2018). Functional hydrogels with tunable structures and properties for tissue engineering applications. Front. Chem. 6, 499. 10.3389/fchem.2018.00499 30406081 PMC6204355

[B35] Li W. Li P. Li N. Du Y. Lü S. Elad D. (2021). Matrix stiffness and shear stresses modulate hepatocyte functions in a fibrotic liver sinusoidal model. Am. J. Physiol. Gastrointest. Liver Physiol. 320, G272–G282. 10.1152/AJPGI.00379.2019 33296275 PMC8609567

[B36] Lim A. Y. Kato Y. Sakolish C. Valdiviezo A. Han G. Bajaj P. (2023). Reproducibility and robustness of a liver microphysiological system PhysioMimix LC12 under varying culture conditions and cell type combinations. Bioengineering 10, 1195. 10.3390/bioengineering10101195 37892925 PMC10603899

[B37] Liu H. Wang Y. Cui K. Guo Y. Zhang X. Qin J. (2019). Advances in hydrogels in organoids and organs-on-a-chip. Adv. Mater. 31, e1902042. 10.1002/adma.201902042 31282047

[B38] Lübberstedt M. Müller-Vieira U. Mayer M. Biemel K. M. Knöspel F. Knobeloch D. (2011). HepaRG human hepatic cell line utility as a surrogate for primary human hepatocytes in drug metabolism assessment *in vitro* . J. Pharmacol. Toxicol. Methods 63, 59–68. 10.1016/j.vascn.2010.04.013 20460162

[B39] Malakpour-Permlid A. Rodriguez M. M. Zór K. Boisen A. Oredsson S. (2025). Advancing humanized 3D tumor modeling using an open access xeno-free medium. Front. Toxicol. 7, 1529360. 10.3389/ftox.2025.1529360 40206700 PMC11979229

[B40] Mas-Bargues C. Sanz-Ros J. Román-Domínguez A. Inglés M. Gimeno-Mallench L. El Alami M. (2019). Relevance of oxygen concentration in stem cell culture for regenerative medicine. Int. J. Mol. Sci. 20, 1195. 10.3390/ijms20051195 30857245 PMC6429522

[B41] McMurtrey R. J. (2016). Analytic models of oxygen and nutrient diffusion, metabolism dynamics, and architecture optimization in three-dimensional tissue constructs with applications and insights in cerebral organoids. Tissue Eng. C Methods 22, 221–249. 10.1089/TEN.TEC.2015.0375 26650970 PMC5029285

[B42] Mendonca T. Lis-Slimak K. Matheson A. B. Smith M. G. Anane-Adjei A. B. Ashworth J. C. (2023). OptoRheo: simultaneous *in situ* micro-mechanical sensing and imaging of live 3D biological systems. Commun. Biol. 6, 463. 10.1038/s42003-023-04780-8 37117487 PMC10147656

[B43] Meng Y. Eshghi S. Li Y. J. Schmidt R. Schaffer D. V. Healy K. E. (2010). Characterization of integrin engagement during defined human embryonic stem cell culture. FASEB J. 24, 1056–1065. 10.1096/FJ.08-126821 19933311 PMC2845424

[B44] Mimetas B. V. (2022). OrganoPlate ® 3-lane 40. Available online at: https://www.mimetas.com/files/products/OrganoPlate%203-lane%2040/202_Flyer_OrganoPlate%203-lane%2040%20product%20specifications.pdf.pdf (Accessed November 29, 2022).

[B46] Nawaz M. Shah N. Zanetti B. R. Maugeri M. Silvestre R. N. Fatima F. (2018). Extracellular vesicles and matrix remodeling enzymes: the emerging roles in extracellular matrix remodeling, progression of diseases and tissue repair. Cells 7, 167. 10.3390/cells7100167 30322133 PMC6210724

[B47] Neves M. I. Moroni L. Barrias C. C. (2020). Modulating alginate hydrogels for improved biological performance as cellular 3D microenvironments. Front. Bioeng. Biotechnol. 8, 665. 10.3389/fbioe.2020.00665 32695759 PMC7338591

[B48] Nitsche K. S. Müller I. Malcomber S. Carmichael P. L. Bouwmeester H. (2022). Implementing organ-on-chip in a next-generation risk assessment of chemicals: a review. Arch. Toxicol. 96, 711–741. 10.1007/s00204-022-03234-0 35103818 PMC8850248

[B49] Nogoceke R. Josino R. Robert A. W. Stimamiglio M. A. (2023). Evaluation of a peptide hydrogel as a chondro-instructive three-dimensional microenvironment. Polymers (Basel) 15, 4630. 10.3390/polym15244630 38139882 PMC10747086

[B50] Novac O. Silva R. Young L.-M. Lachani K. Hughes D. Kostrzewski T. (2022). Human liver microphysiological system for assessing drug-induced liver toxicity *in vitro* . J. Vis. Exp. 2022. 10.3791/63389 35156664

[B51] Ohashi K. Fujiwara S. Mizuno K. (2017). Roles of the cytoskeleton, cell adhesion and rho signalling in mechanosensing and mechanotransduction. J. Biochem. 161, 245–254. 10.1093/jb/mvw082 28082721

[B52] Oredsson S. Coecke S. van der Valk J. Vinken M. (2019). What is understood by “animal-free research”. Toxicol. Vitro 57, 143–144. 10.1016/J.TIV.2019.03.001 30849472

[B53] Ott L. M. Ramachandran K. Stehno-Bittel L. (2017). An automated multiplexed hepatotoxicity and CYP induction assay using HepaRG cells in 2D and 3D. SLAS Discov. 22, 614–625. 10.1177/2472555217701058 28346810

[B54] Pal R. Mamidi M. K. Das A. K. Bhonde R. (2012). Diverse effects of dimethyl sulfoxide (DMSO) on the differentiation potential of human embryonic stem cells. Arch. Toxicol. 86, 651–661. 10.1007/s00204-011-0782-2 22105179

[B55] Pamies D. Ekert J. Zurich M. G. Frey O. Werner S. Piergiovanni M. (2024). Recommendations on fit-for-purpose criteria to establish quality management for microphysiological systems and for monitoring their reproducibility. Stem Cell Rep. 19, 604–617. 10.1016/j.stemcr.2024.03.009 38670111 PMC11103889

[B56] Pfeifer L. M. Sensbach J. Pipp F. Werkmann D. Hewitt P. (2024). Increasing sustainability and reproducibility of *in vitro* toxicology applications: serum-free cultivation of HepG2 cells. Front. Toxicol. 6, 1439031. 10.3389/ftox.2024.1439031 39650261 PMC11621109

[B57] Phiwchai I. Thongtem T. Thongtem S. Pilapong C. (2021). Liver cancer cells uptake labile iron *via* L-type calcium channel to facilitate the cancer cell proliferation. Cell Biochem. Biophys. 79, 133–139. 10.1007/s12013-020-00951-0 33064258

[B58] Proença S. Escher B. I. Fischer F. C. Fisher C. Grégoire S. Hewitt N. J. (2021). Effective exposure of chemicals in *in vitro* cell systems: a review of chemical distribution models. Toxicol. Vitro 73, 105133. 10.1016/j.tiv.2021.105133 33662518

[B59] Punt A. Peijnenburg A. A. C. M. Hoogenboom R. L. A. P. Bouwmeester H. (2017). Non-animal approaches for toxicokinetics in risk evaluations of food chemicals. ALTEX 34, 501–514. 10.14573/altex.1702211 28403478

[B60] QYResearch (2024). Global and United States Matrigel basement membrane matrix market report & forecast 2024- 2030. Available online at: https://www.qyresearch.com/reports/3152845/matrigel-basement-membrane-matrix (Accessed October 24, 2024).

[B61] Rafnsdóttir Ó. B. Kiuru A. Tebäck M. Friberg N. Revstedt P. Zhu J. (2023). A new animal product free defined medium for 2D and 3D culturing of normal and cancer cells to study cell proliferation and migration as well as dose response to chemical treatment. Toxicol. Rep. 10, 509–520. 10.1016/j.toxrep.2023.04.001 37396848 PMC10313884

[B62] Ramaiahgari S. C. Ferguson S. S. (2019). Organotypic 3D HepaRG liver model for assessment of drug-induced cholestasis. Methods Mol. Biol. 1981, 313–323. 10.1007/978-1-4939-9420-5_20 31016663

[B63] Ramaiahgari S. C. Waidyanatha S. Dixon D. DeVito M. J. Paules R. S. Ferguson S. S. (2017). From the cover: three-dimensional (3D) HepaRG spheroid model with physiologically relevant xenobiotic metabolism competence and hepatocyte functionality for liver toxicity screening. Toxicol. Sci. 159, 124–136. 10.1093/toxsci/kfx122 28633424 PMC5837526

[B64] Rebelo S. P. Costa R. Estrada M. Shevchenko V. Brito C. Alves P. M. (2015). HepaRG microencapsulated spheroids in DMSO-free culture: novel culturing approaches for enhanced xenobiotic and biosynthetic metabolism. Arch. Toxicol. 89, 1347–1358. 10.1007/s00204-014-1320-9 25107451

[B65] Ribeiro A. J. S. Yang X. Patel V. Madabushi R. Strauss D. G. (2019). Liver microphysiological systems for predicting and evaluating drug effects. Clin. Pharmacol. Ther. 106, 139–147. 10.1002/CPT.1458 30993668 PMC6771674

[B66] Rusyn I. Sakolish C. Kato Y. Stephan C. Vergara L. Hewitt P. (2022). Microphysiological systems evaluation: experience of TEX-VAL tissue chip testing consortium. Toxicol. Sci. 188, 143–152. 10.1093/toxsci/kfac061 35689632 PMC9333404

[B67] Singh B. Abdelgawad M. E. Ali Z. Bailey J. Budyn E. Civita P. (2021). Towards more predictive, physiological and animal-free *in vitro* models. Adv. Cell Tissue Cult. 2020 Conf. Proc. *Altern. Laboratory Animals* 49, 93–110. 10.1177/02611929211025006 34225465

[B68] Solbu A. A. Caballero D. Damigos S. Kundu S. C. Reis R. L. Halaas Ø. (2023). Assessing cell migration in hydrogels: an overview of relevant materials and methods. Mater. Today Bio. 18, 100537. 10.1016/j.mtbio.2022.100537 36659998 PMC9842866

[B69] Stanley L. A. Wolf C. R. (2022). Through a glass, darkly? HepaRG and HepG2 cells as models of human phase I drug metabolism. Drug Metab. Rev. 54, 46–62. 10.1080/03602532.2022.2039688 35188018

[B70] Sun W. Zhang S. Zhou T. Shan Y. Gao F. Zhang Y. (2020). Human urinal cell reprogramming: synthetic 3D peptide hydrogels enhance induced pluripotent stem cell population homogeneity. ACS Biomater. Sci. Eng. 6, 6263–6275. 10.1021/acsbiomaterials.0c00667 33449655

[B71] Talbot N. C. Caperna T. J. (2015). Proteome array identification of bioactive soluble proteins/peptides in Matrigel: relevance to stem cell responses. Cytotechnology 67, 873–883. 10.1007/s10616-014-9727-y 24744128 PMC4545444

[B72] Tao T. P. Brandmair K. Gerlach S. Przibilla J. Géniès C. Jacques-Jamin C. (2021). Demonstration of the first-pass metabolism in the skin of the hair dye, 4-amino-2-hydroxytoluene, using the Chip2 skin–liver microphysiological model. J. Appl. Toxicol. 41, 1553–1567. 10.1002/jat.4146 33594739

[B73] Teng Y. Zhao Z. Tasnim F. Huang X. Yu H. (2021). A scalable and sensitive steatosis chip with long-term perfusion of *in situ* differentiated HepaRG organoids. Biomaterials 275, 120904. 10.1016/j.biomaterials.2021.120904 34119888

[B74] van der Valk J. Bieback K. Buta C. Cochrane B. Dirks W. G. Fu J. (2018). Fetal bovine serum (FBS): past – present – future. ALTEX 35, 99–118. 10.14573/altex.1705101 28800376

[B75] van Wenum M. Adam A. A. A. van der Mark V. A. Chang J. C. Wildenberg M. E. Hendriks E. J. (2018). Oxygen drives hepatocyte differentiation and phenotype stability in liver cell lines. J. Cell Commun. Sig. 12, 575–588. 10.1007/s12079-018-0456-4 29399736 PMC6039343

[B76] Waizenegger J. Glück J. Henricsson M. Luckert C. Braeuning A. Hessel-Pras S. (2021). Pyrrolizidine alkaloids disturb bile acid homeostasis in the human hepatoma cell line HepaRG. Foods 10, 161–16. 10.3390/foods10010161 33466663 PMC7828834

[B77] Wang J. B. Qin W. Yang Z. Shen S. Ma Y. Wang L. Y. (2022). Optimization of three-dimensional culture conditions of HepG2 cells with response surface methodology based on the VitroGel system. Biomed. Environ. Sci. 35, 688–698. 10.3967/bes2022.091 36127781

[B78] Weber T. Wiest J. Oredsson S. Bieback K. (2022). Case studies exemplifying the transition to animal component-free cell culture. Altern. Laboratory Animals 50, 330–338. 10.1177/02611929221117999 35983799

[B79] Winter S. J. Miller H. A. Steinbach-Rankins J. M. (2021). Multicellular ovarian cancer model for evaluation of nanovector delivery in ascites and metastatic environments. Pharmaceutics 13, 1891. 10.3390/pharmaceutics13111891 34834307 PMC8625169

[B80] Wu Q. Liu J. Wang X. Feng L. Wu J. Zhu X. (2020). Organ-on-a-chip: recent breakthroughs and future prospects. Biomed. Eng. Online 19, 9. 10.1186/s12938-020-0752-0 32050989 PMC7017614

[B81] Yan H. J. Casalini T. Hulsart-Billström G. Wang S. Oommen O. P. Salvalaglio M. (2018). Synthetic design of growth factor sequestering extracellular matrix mimetic hydrogel for promoting *in vivo* bone formation. Biomaterials 161, 190–202. 10.1016/j.biomaterials.2018.01.041 29421555

[B82] Zeiringer S. Wiltschko L. Glader C. Reiser M. Absenger-Novak M. Fröhlich E. (2023). Development and characterization of an *in vitro* intestinal model including extracellular matrix and macrovascular endothelium. Mol. Pharm. 20, 5173–5184. 10.1021/acs.molpharmaceut.3c00532 37677739 PMC10548470

