# Supplementary material for: Alternatives to animal-derived extracellular matrix hydrogels? An explorative study with HepaRG cells in animal-free hydrogels under static and dynamic culture conditions
Source: Front Toxicol. 2025 Oct 30;7:1649393. doi: 10.3389/ftox.2025.1649393 (PMC12611971; doi:10.3389/ftox.2025.1649393)
Supplement: Supplementary file 1 [file Supplementaryfile1.docx]

Supplementary information

Alternatives to animal-derived extracellular matrix hydrogels? An explorative study with HepaRG cells in animal-free hydrogels under static and dynamic culture conditions

Katharina S. Nitsche¹*, Paul L. Carmichael¹ ², Sophie Malcomber², Iris Mueller² & Hans Bouwmeester¹


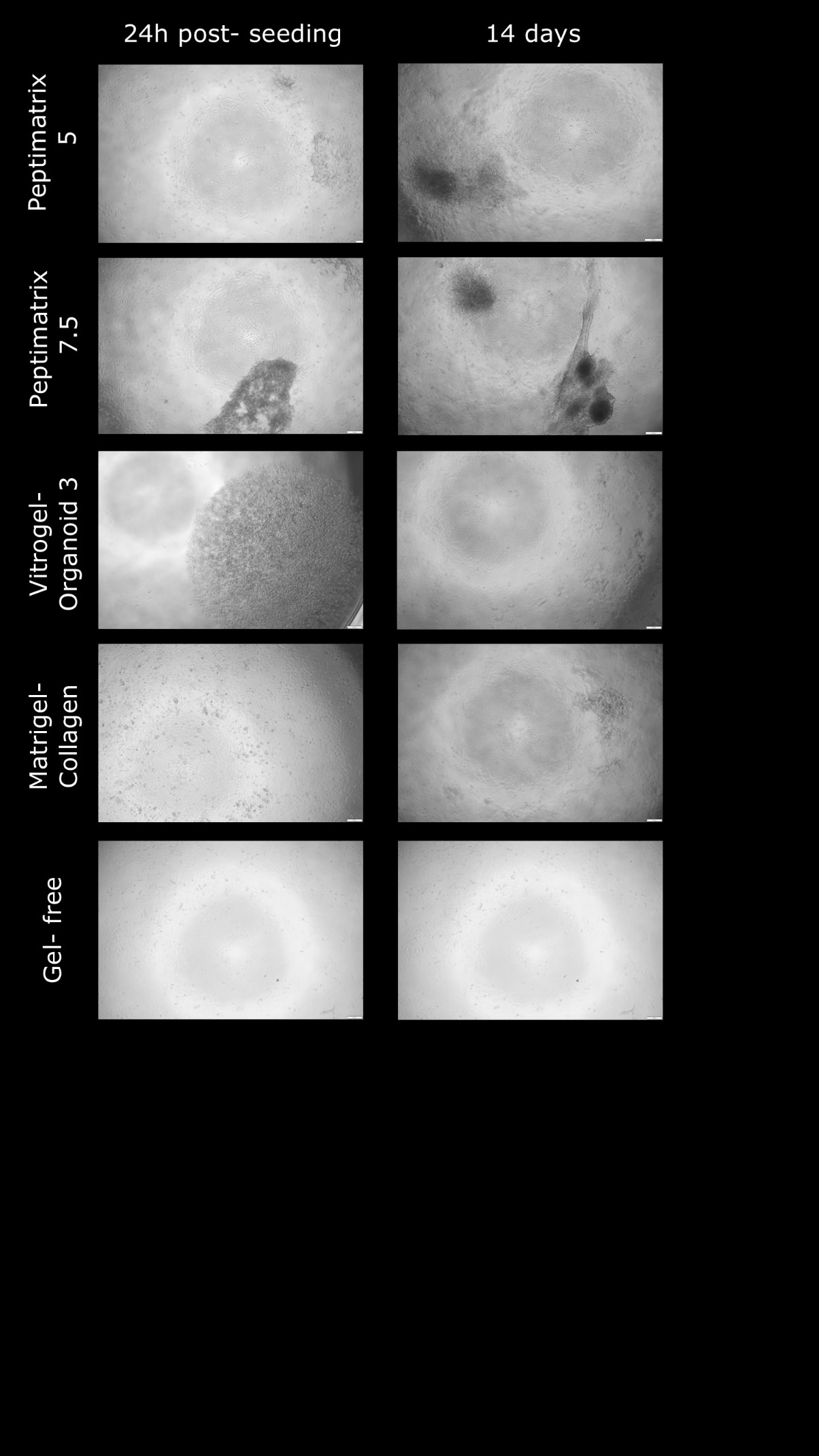


Figure SI. 1 Analysis of HepaRG cell distribution in Peptimatrix 5 and 7.5 (PeptiMatrix™), Vitrogel Organoid 3 (The Well Bioscience) and Matrigel-Collagen (Corning Inc and R&D Systems) on the 96 well plate (n=3). 10x Microscopic pictures of HepaRG cells 24 hours and 14 days post-seeding , illustrating differences in cell distribution and attachment


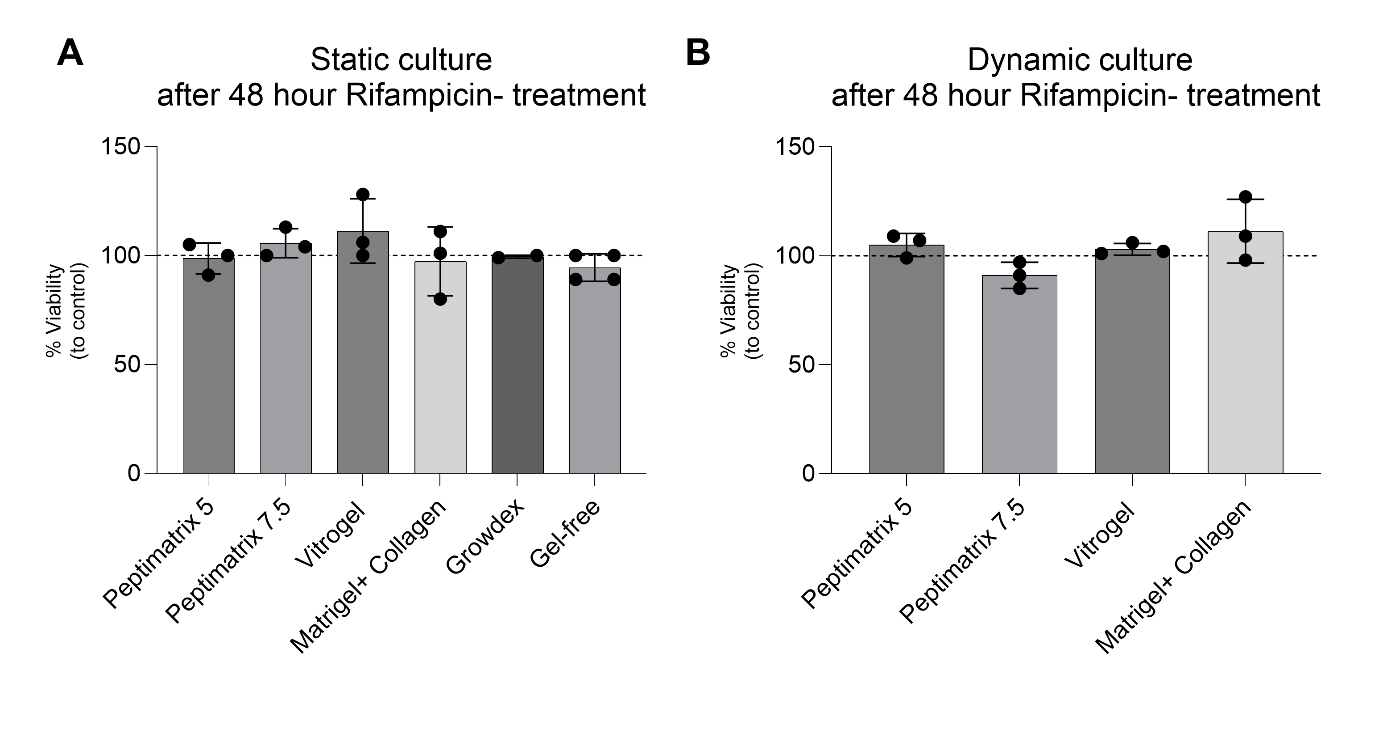


Figure SI. 2 Cell viability of HepaRG cells upon 48 h treatment with 25 µM Rifampicin, assessed using the WST-8 assay. Values represent the mean ± SD. Treatment effects were assessed with a multiple unpaired t test with Welch correction (n=3)


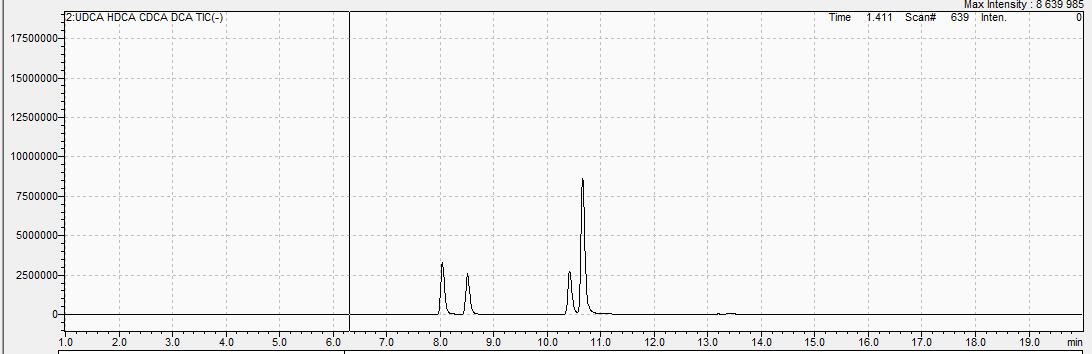


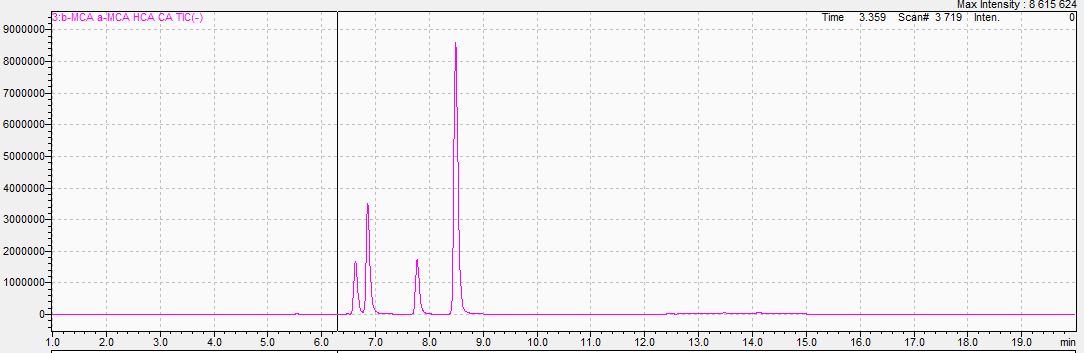


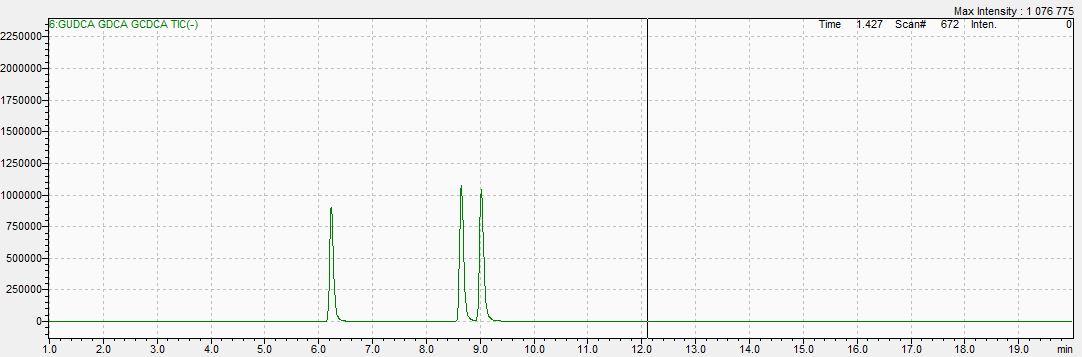


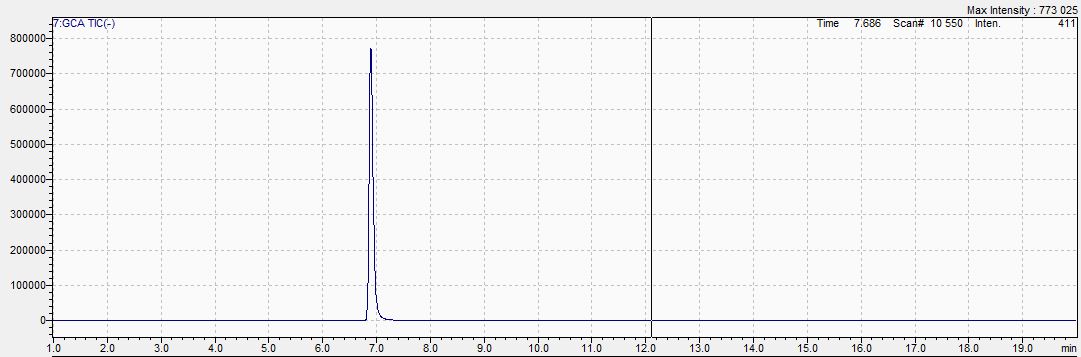


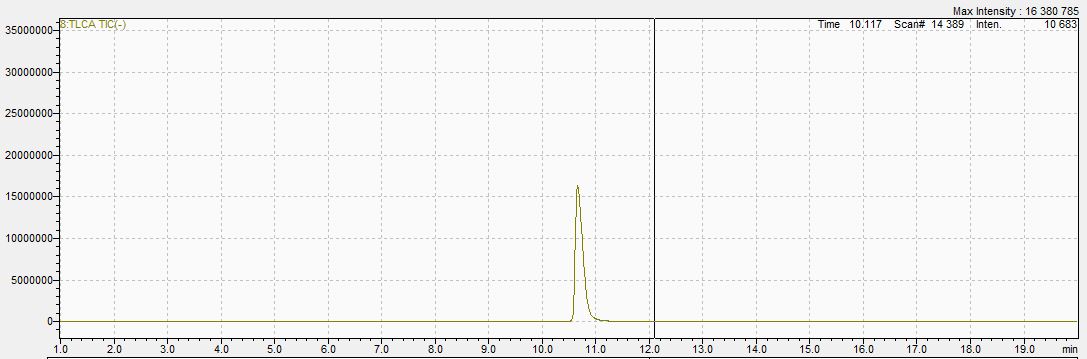


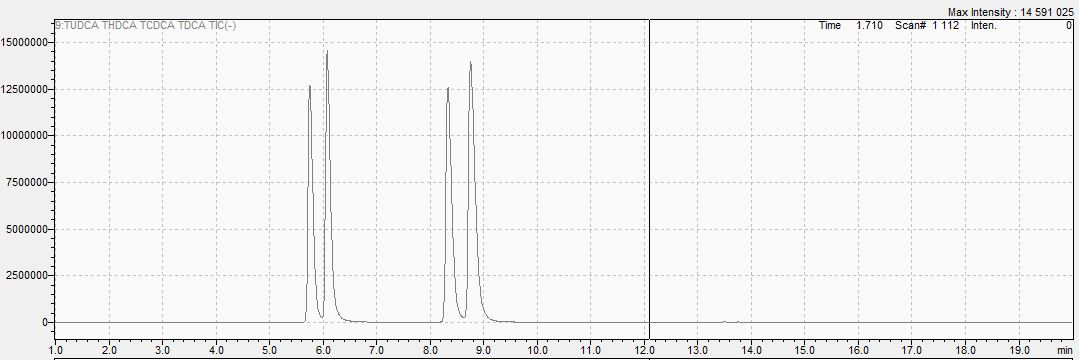


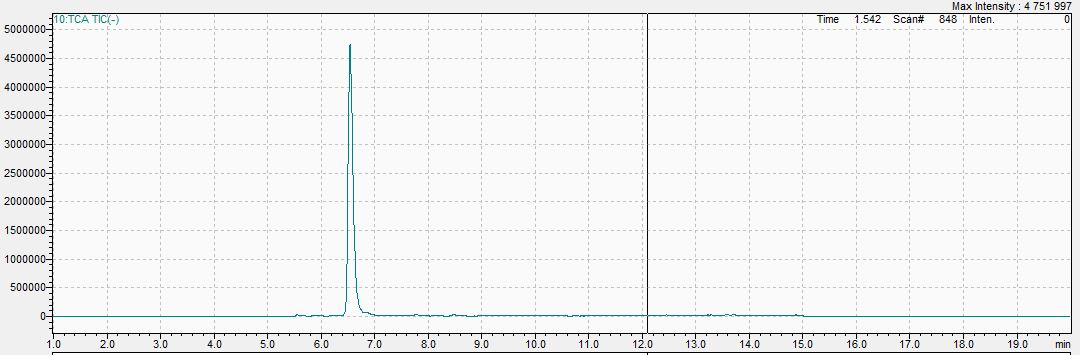


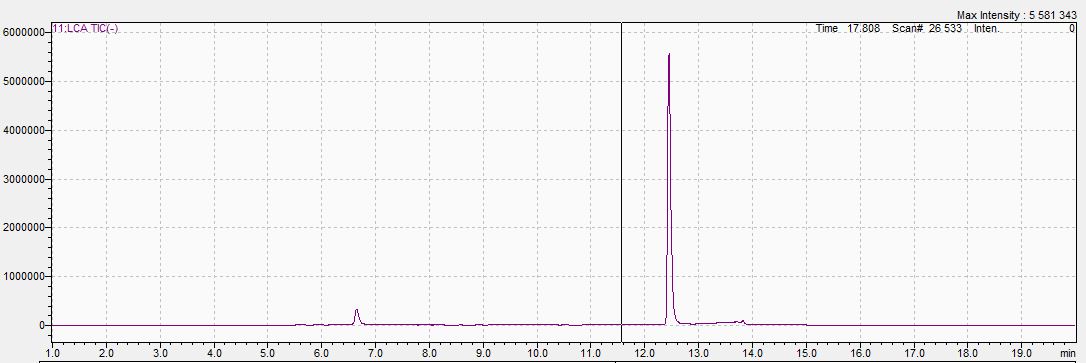


Figure SI. 3 Chromatograms of LC-MS/MS run with 10 μM mixture of 18 BAs in MeOH. Abbreviations, MS parameters and LOD are presented in Table SI. 1.


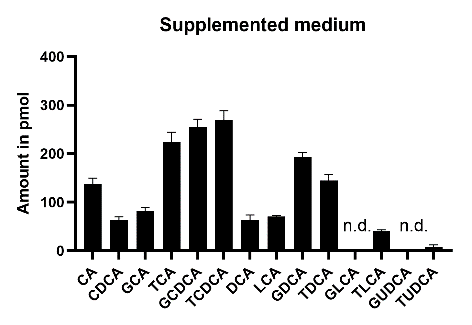


Figure SI. 4 Bile acid levels in supplemented medium used for static and dynamic culture. n.d.= not detected


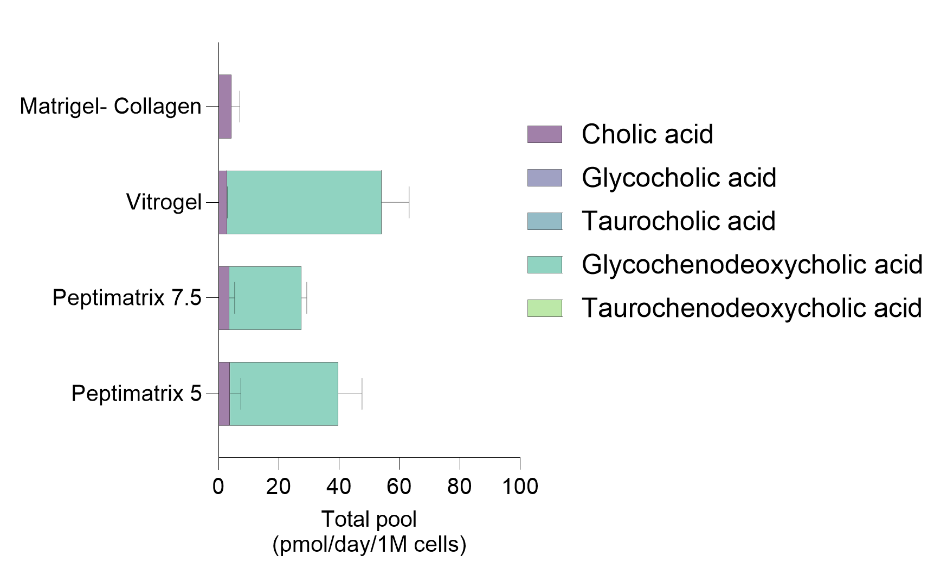


Figure SI. 5 **Quantification of primary (conjugated) bile acid secretion in pooled cell culture medium of static cultured HepaRG on day 14**. Values present the mean ±SD of six pooled wells normalised with initial seeding cell count and corrected for bile acid amounts present in supplemented culture medium (see Supplementary Figure SI. 4). The concentration of the remaining 16 BAs was below the limit of quantification (see Supplementary Table SI. 1 for limits of detection (LOD) and quantification). Statistical differences between hydrogels were determined with an ordinary one-way ANOVA with Tukeys multiple comparison and Brown-Forsythe test ( n=3)

.Table SI. 1 MS parameters, limit of detection (LOD) and limit of quantification (LOQ) of the studied BAs

|  | **Mode** | **Q1** | **Q3** | **Retention time (min)** | **LOD (µM) in MeOH** | **LOQ (µM) in MeOH** |
| --- | --- | --- | --- | --- | --- | --- |
| **Lithocholic acid (LCA)** | SIM |  | 375.3 | 13.12 | 0.01 | 0.05 |
| **Ursodeoxycholic acid (UDCA)** | SIM |  | 391.3 | 8.113 | 0.05 | 0.1 |
| **Hyodeoxycholic acid (HDCA)** | SIM |  | 391.3 | 8.725 | 0.05 | 0.1 |
| **Chenodeoxycholic acid (CDCA)** | SIM |  | 391.3 | 10.809 | 0.05 | 0.1 |
| **Deoxycholic acid (DCA)** | SIM |  | 391.3 | 11.073 | 0.1 | 0.5 |
| **Hyocholic acid (HCA)** | SIM |  | 407.3 | 8.145 | 0.01 | 0.05 |
| **Cholic acid (CA)** | SIM |  | 407.3 | 8.624 | 0.005 | 0.01 |
| **Glycolithocholic acid (GLCA)** | MRM | 432.3 | 74 | 11.007 | 0.01 | 0.05 |
| **Glycoursodeoxycholic acid (GUDCA)** | MRM | 448.3 | 74 | 6.295 | 0.05 | 0.1 |
| **Glycochenodeoxycholic acid (GCDCA)** | MRM | 448.3 | 74 | 9.051 | 0.001 | 0.005 |
| **Glycodeoxycholic acid (GDCA)** | MRM | 448.3 | 74 | 9.063 | 0.05 | 0.1 |
| **Glycocholic acid (GCA)** | MRM | 464.3 | 74 | 6.998 | 0.05 | 0.1 |
| **Taurolithocholic acid (TLCA)** | SIM |  | 482.3 | 11.077 | 0.1 | 0.5 |
| **Tauroursodeoxycholic acid (TUDCA)** | SIM |  | 498.4 | 5.911 | 0.05 | 0.1 |
| **Taurohyodeoxycholic acid (THDCA)** | SIM |  | 498.4 | 6.425 | 0.05 | 0.1 |
| **Taurochenodeoxycholic acid (TCDCA)** | SIM |  | 498.4 | 8.825 |  |  |
| **Taurodeoxycholic acid (TDCA)** | SIM |  | 498.4 | 9.283 |  |  |
| **Taurocholic acid (TCA)** | SIM |  | 514.4 | 7.001 |  |  |

SIM: Single Ion Monitoring; MRM: Multiple Reaction Monitoring; Q1: first stage MS, Q3: second stage MS; LOD: limit of detection; LOQ: limit of quantification

Table SI. 2 List of primers used in the RT-qPCR analysis

| Protein name | Gene Symbol | Forward Primer | Reverse Primer | Remarks |
| --- | --- | --- | --- | --- |
| Albumin | ALB | TGCAACTCTTCGTGAAACCTATG | ACATCAACCTCTGGTCTCACC |  |
| Cytochrome P450 3A4 | CYP3A4 | AAGTCGCCTCGAAGATACACA | AAGGAGAGAACACTGCTCGTG |  |
| Cytochrome P450 27A1 | CYP27A1 | AGGCCAAGTACGGTCCAATG | GTACCAGTGGTGTCCTTCCG |  |
| Cytochrome P450 7B1 | CYP7B1 | ATGCAAGGATGGTCAGAAGTTTT | TGGGTGCCGCAGAAGATAATA |  |
| Cytokeratin 18 | KRT18 | ACCAAGTTTGAGACGGAACAG | CCCTCAGCGTACTGATTTCCT |  |
| Cytokeratin 19 | KRT19 | GGCATCCAGAACGAGAAGGAG | ATTGTCCACAGTATTTGCGAAGA |  |
| TATA-binding protein | TBP | GAGCCAAGAGTGAAGAACAGTC | GCTCCCCACCATATTCTGAATCT | House-keeping gene |
| Succinate dehydrogenase complex flavoprotein subunit A | SDHA | CAAACAGGAACCCGAGGTTTT | CAGCTTGGTAACACATGCTGTAT | House-keeping gene |
| ubiquitin C | UBC | GGAGCCGAGTGACACCATTG | CAGGGTACGACCATCTTCCAG | House-keeping gene |
